# Alzheimer’s Disease Pathology in Middle Aged and Older People with HIV: Comparisons with Non-HIV Controls on a Healthy Aging and Alzheimer’s Disease Trajectory and Relationships with Cognitive Function

**DOI:** 10.3390/v15061319

**Published:** 2023-06-04

**Authors:** Erin E. Sundermann, Laura M. Campbell, Olivia Villers, Mark W. Bondi, Ben Gouaux, David P. Salmon, Douglas Galasko, Virawudh Soontornniyomkij, Ronald J. Ellis, David J. Moore

**Affiliations:** 1 Department of Psychiatry, University of California San Diego, 9500 Gilman Dr., La Jolla, CA 92093, USAdjmoore@health.ucsd.edu (D.J.M.); 2 San Diego State University/University of California San Diego Joint Doctoral Program in Clinical Psychology, 6363 Alvarado Court, Suite 103, San Diego, CA 92120, USA; 3 School of Medicine, University of California San Diego, 9500 Gilman Dr., La Jolla, CA 92093, USA; 4 VA San Diego Healthcare System, 3350 La Jolla Village Dr., San Diego, CA 92161, USA; 5 Department of Neurosciences, University of California San Diego, 9375 Gilman Dr., La Jolla, CA 92161, USA

**Keywords:** HIV, Alzheimer’s disease, pathology, amyloid-beta, tau, cognition, sex

## Abstract

We determined the prevalence of Alzheimer’s disease (AD) pathological hallmarks, amyloid-β and phosphorylated-Tau, in autopsied brains of 49 people with HIV (PWH) (ages: 50–68; mean age = 57.0) from the National NeuroAIDS Tissue Consortium and in a comparative cohort of 55 people without HIV (PWoH) from the UC San Diego Alzheimer’s Disease Research Center (17 controls, 14 mild cognitive impairment, 24 AD; ages: 70–102, mean age = 88.7). We examined how AD pathology relates to domain-specific cognitive functions in PWH overall and in sex-stratified samples. Amyloid-β and phosphorylated-Tau positivity (presence of pathology of any type/density) was determined via immunohistochemistry in AD-sensitive brain regions. Among PWH, amyloid-β positivity ranged from 19% (hippocampus) to 41% (frontal neocortex), and phosphorylated-Tau positivity ranged from 47% (entorhinal cortex) to 73% (transentorhinal cortex). Generally, AD pathology was significantly less prevalent, and less severe when present, in PWH versus PWoH regardless of cognitive status. Among PWH, positivity for AD pathology related most consistently to memory-related domains. Positivity for p-Tau pathology related to memory-related domains in women with HIV only, although the sample size of women with HIV was small (*n* = 10). Results indicate that AD pathology is present in a sizable portion of middle aged and older PWH, although not to the extent in older PWoH. Studies with better age-matched PWoH are needed to examine the effect of HIV status on AD pathology.

## 1. Introduction

People with human immunodeficiency virus (PWH) are living longer due to effective antiretroviral therapy (ART). Currently, about one half of PWH in the United States are age 50 and older, and 24% of PWH are age 60 and older, with this rate rising steadily [1]. As such, the risk of age-associated neurodegenerative disorders including Alzheimer’s disease (AD) and its precursor, amnestic mild cognitive impairment (aMCI), is a concern for older PWH. PWH may be more susceptible to AD and/or earlier AD onset due to shared biological mechanisms between AD and HIV. These shared mechanisms include low-grade inflammation, metabolic dysregulation, oxidative stress, and cardiovascular disease, all of which raise the potential for the compounding effects of HIV and aging on the brain [2,3,4,5]. Evidence of premature or accelerated aging among PWH is reflected molecularly, with brain tissue revealing more advanced epigenetic age by 7.4 years in PWH compared to people without HIV (PWoH), and epidemiologically by the earlier appearance of age-associated conditions in PWH versus the general population by 5–10 years [6,7,8]. It is unclear whether evidence of accelerated aging among PWH extends to the development of AD-related pathology.

Neuropathologic diagnosis of AD requires amyloid beta (Aβ) plaques, especially cored neuritic plaques, and neurofibrillary tangles identified by methods such as antibodies against phosphorylated-Tau (p-Tau). Aβ plaques initially appear in the cerebral neocortex whereas cerebral p-Tau pathology originates in the transentorhinal cortex, and both of these pathologies subsequently progress to involve other brain regions in characteristic sequences [9,10]. Both pathologies are observed in normal aging; however, excessive burden is indicative of AD with the development of both pathologies, typically predating the appearance of clinical symptoms by a decade or more [11,12].

Aβ plaques have been observed in the brains of PWH in postmortem studies, particularly in middle aged and older (e.g., age 50+) cases and those with cognitive impairment [13,14,15,16,17,18,19,20]. A similar regional distribution to that found in healthy aging and AD, namely, the mid-temporal and frontal lobe regions, has been reported [14]. In a postmortem study comparing Aβ plaque prevalence in a cohort of PWH (*n* = 273; age range: 31–70) to rates reported on by Braak et al. in an age-matched cohort in the general population, Soontornniyomkij et al. (2019) found that the frequency of Aβ plaque-bearing cases was slightly higher in the PWH cohort versus the general population cohort (29.3% versus 25.8%), and that the regional/spatial initiation and progression of Aβ plaque deposition are similar regardless of HIV status [20]. Others have reported greater intracellular Aβ plaque pathology burden in PWH compared to age-matched PWoH in postmortem studies [13,14,18,21], although not consistently [19,22,23]. In contrast, positron emission tomography (PET) studies have consistently reported no evidence of elevated extracellular amyloid fibrillar deposits in PWH with or without cognitive impairment [24,25,26]; however, the sample size is small in these studies and the PET tracer, Pittsburgh Compound B, measures cored extracellular fibrillar deposits, whereas the Aβ plaques typically observed in healthy aging and in PWH are diffuse [16,27,28,29]. Research regarding Tau pathology in PWH is more limited than that pertaining to Aβ. P-Tau immunostaining has been observed in the frontal cortex, hippocampus, putamen, and basal neocortex of PWH in postmortem studies [20,30,31], with a strong positive association to Aβ pathology burden [19,32]. An elevated p-Tau pathology burden has been demonstrated in the brains of PWH on ART when compared to PWoH [31], though p-Tau lesions were found to be sparse and vary greatly by brain region [20,31]. On the other hand, studies examining cerebrospinal fluid (CSF) levels of p-Tau reported no difference between PWH and PWoH [33,34,35,36], although inconsistencies do exist [37]. Similarly, PET studies investigating p-Tau pathology reported similar p-Tau pathology burden in PWH and PWoH [38]. Even fewer studies have examined how p-Tau pathology relates to cognitive function among PWH. Soontornniyomkij et al. (2019) reported that regional p-Tau lesions did not significantly relate to cognitive function across domains [20].

The potential for sex disparities in the prevalence of AD pathology and the link between AD pathology and clinical symptoms in PWH is an important unanswered question given well-established sex differences in rates of HIV-associated cognitive impairment and AD [39,40], AD pathology burden [41,42,43,44,45,46], and in the clinical manifestation of AD pathology [41,47,48]. Women represent two-thirds of AD cases in the U.S. [49] and multiple epidemiological studies indicate higher age-specific incidence rates in women as well [50,51,52]. Several studies have also indicated that women tend to show a greater burden of AD pathology, particularly of p-Tau [41,42,43,44,45,46], and more rapid cognitive decline than men in the mild cognitive impairment (MCI) stage [53,54,55]. In the post-mortem Religious Orders Study, each additional unit of AD pathology was associated with a 3-fold increase in the likelihood of clinical AD in men compared with a 20-fold increased likelihood in women [56]. Among PWH, a meta-analysis and systematic review have also indicated a higher rate of HIV-associated cognitive impairment in women versus men as well as differing profiles of HIV-associated cognitive impairment [39,40]. This current study represents the first to investigate how the burden of AD pathology and its clinical significance differ between women versus men with HIV.

Most prior studies examining Aβ and p-Tau burden in PWH were not restricted to middle aged to older PWH, leaving the question of the prevalence of Aβ and p-Tau pathology in the brain tissue of this group unanswered. Additionally, it is unclear how the level of AD pathology in PWH compares to that of PWoH on an AD trajectory, and how this pathology impacts cognitive function. Some studies have reported a relationship between greater Aβ deposition and poorer cognitive outcomes among PWH [20,57], but have rarely conducted a domain-specific analysis and have not examined sex differences. Addressing the specific cognitive domains associated with AD pathology among PWH is a critical step to determine the significance of this pathology to AD risk in PWH, as the cognitive presentation of early AD tends to show domain specificity given that memory deficits are typically regarded as the earliest and most salient symptom of AD [58,59]. The present study aims to address these knowledge gaps by assessing the prevalence of Aβ and p-Tau pathology across AD-sensitive brain regions, such as the prefrontal neocortex, putamen, basal-temporal neocortex, and hippocampus, among middle aged to older (age 50+ years) postmortem PWH from the National NeuroAIDS Tissue Consortium (NNTC). To provide context as to the typical levels of AD pathology among PWoH on the aging and AD trajectory, we compared Aβ and p-Tau prevalence rates among our PWH cases to normal control, MCI, and AD dementia cases from the UC San Diego Shiley-Marcos Alzheimer’s Disease Research Center (ADRC). To assess the clinical significance of the Aβ and p-Tau pathology in PWH, we examined how the presence of region-specific and overall Aβ and p-Tau pathology relates to antemortem, domain-specific cognitive performance. We hypothesized that the presence of Aβ and p-Tau pathology would relate to the AD-sensitive domains of learning, recall, executive function, and language (verbal fluency), with the learning and recall domains showing the strongest and most consistent relationships. Additionally, we explored how the prevalence of Aβ and p-Tau pathology and the association between pathology and cognition might differ by sex among PWH.

## 2. Materials and Methods

### 2.1. NNTC Cohort

Participants: Postmortem cases of PWH were from the following four sites of the NNTC [60] (www.nntc.org (accessed on 1 November 2022)): University of Texas Medical Branch at Galveston, University of California San Diego, University of California Los Angeles, and Mount Sinai Medical Center in New York. The sample was limited to those at least 50 years old at death. Sample size varied by brain region. The largest sample size of 49 consisted of PWH with frontal neocortex Aβ and p-Tau characterization (age range: 50–68; mean age = 57 [SD = 5.0], 20% female, 52% non-Hispanic white; Table 1). Aβ and p-Tau pathology characterization was available for 34 PWH in the basal temporal neocortex and for 32 PWH for the hippocampus. P-Tau characterization was available for 30 PWH for the transentorhinal and entorhinal cortex. Year of death ranged from 1999 to 2013.

Neuromedical Evaluation: NNTC participants completed a standardized neuromedical evaluation within a year of death. DSM-IV diagnoses of current and lifetime alcohol and other substance use disorders (amphetamine, cannabis, cocaine, hallucinogens, inhalant, sedatives, opioids and PCP) and major depressive disorder (MDD) were determined based on the fully structured computer-based Composite International Diagnostic Interview version 2.1 (WHO, 1997). History of antemortem medical comorbidities (e.g., hypertension, diabetes, hyperlipidemia) was available for 36 participants and was determined by self-report or self-reported medication records. Genotyping of the apolipoprotein-E ε4 (*APOE*-ε4) allele, the strongest genetic risk factor for sporadic AD, was conducted for all participants and is described elsewhere [21]. HIV disease characteristics were determined either by self-report or laboratory testing. Estimated duration of HIV disease was self-reported. Current ART use was self-reported and available in 40 participants. Nadir CD4+ T-cell count was the lowest lifetime value among self-report and study obtained CD4+ T-cell counts and released medical records, and was available in 31 participants. Antemortem CD4+ T-cell count was measured with flow cytometry. Antemortem plasma HIV-1 RNA level was measured by ultra-sensitive PCR in a CLIA-certified clinical laboratory, and viral suppression was defined as an HIV-1 RNA level below the lower limit of quantification of 50 copies/mL (Amplicor, Roche Diagnostic System).

Neuropsychological Evaluation: Scores on a standardized neurocognitive test battery conducted within eighteen months of death were available for most PWH (*n* = 41–45). The cognitive domains assessed include: verbal fluency, working memory, speed of information processing, learning and delayed recall, executive function, and complex motor function. Scores on the Wide Range Achievement Test-3 Reading subtest (WRAT-3), a proxy for cognitive reserve, were also available for 46 PWH and presented for sample description purposes. Specific tests are described elsewhere [61]. Raw test scores were transformed into demographically-adjusted (i.e., age, education, sex, and race/ethnicity) T-scores based on normative samples of non-HIV participants [62,63]. T-scores were averaged across tests within domains to obtain domain-specific T-scores [64,65,66]. The sample size varied slightly by domain T-score and were as follows: 45 with learning and memory T-scores, 44 with attention/working memory and verbal fluency T-scores, 43 with executive function T-scores, 42 with speed of information processing T-scores, and 41 with complex motor function T-scores.

### 2.2. ADRC Cohort

Participants: Postmortem cases of PWoH were from the UC San Diego ADRC and included 55 cases (17 normal control, 14 MCI and 24 AD dementia) with data available from a neuropathological evaluation and an antemortem clinical assessment within a year of death (age range: 70–102, mean age = 88.7 [SD = 7.04], 53% female, 96.7% non-Hispanic White). Among cases with available frozen brain tissue in the ADRC repository, we prioritized those that: (1) were younger in order to more closely approximate the age distribution of the NNTC, (2) were diagnosed at their antemortem study visit as normal control (NC), aMCI or AD dementia, and (3) allowed for a more equitable distribution of NC, MCI and AD dementia cases. Among the AD dementia cases, 96% had confirmed AD neuropathologic changes at autopsy, 80% of which were of sufficient severity for a pathological diagnosis of AD.

Clinical Diagnosis: As part of the standard ADRC research protocol, participants completed annual clinical, neurologic, and neuropsychological evaluations [67,68]. Up to 19 cognitive tests that measured the cognitive domains of Learning/Memory, Executive Function, Attention, Visuospatial Function, and Language were administered. A diagnosis of NC, MCI, or dementia was determined at each visit by the consensus of a multidisciplinary team consisting of two senior neurologists and a neuropsychologist based on the *Diagnostic and Statistical Manual of Mental Disorders*, 5th Edition and National Institute on Aging-Alzheimer’s Association (NIA-AA) diagnostic criteria for AD dementia and for MCI.

Neuromedical Evaluation: The history of antemortem medical comorbidities was assessed via a clinical interview and physical examination at their last visit prior to death, and these data were available in 33 of the 55 ADRC participants. The presence or absence of diabetes mellitus, hypertension, and hypercholesterolemia were determined via clinical interview, physical exam, and laboratory tests (e.g., blood glucose and cholesterol levels). A history of MDD and alcohol and substance use diagnoses were obtained via self-report.

### 2.3. Neuropathological Characterization in NNTC and ADRC

In the NNTC cohort of PWH, Aβ plaques and p-Tau lesions were characterized in the brain tissue from the frontal neocortex in 49 participants, in the basal temporal neocortex of 34 of the 49 participants, and in the hippocampus of 32 of the 49 participants. Additionally, p-Tau pathology was characterized in the transentorhinal and entorhinal cortex of 30 of 49 NNTC participants. In the ADRC cohort of PWoH, Aβ and p-Tau lesions were characterized in brain tissue from the frontal neocortex, basal temporal neocortex, and hippocampus, while p-Tau lesions alone were characterized in the transentorhinal and entorhinal cortex in all 53 participants. Brain tissue was extracted as soon as possible after death (maximum120 h post-mortem delay).

Five-μm-thick formalin-fixed paraffin-embedded tissue sections with no significant histopathologic changes were immunohistochemically stained with primary antibodies to Aβ (mouse monoclonal, clone 4G8, #SIG-39220, Covance, Princeton, NJ, USA, 1:20,000 dilution) and p-Tau (mouse monoclonal, clone AT8, #MN1020, Pierce Biotechnology, Rockford, IL, USA, 1:1000). For antigen retrieval, the sections were incubated with 88% formic acid (5 min for Aβ staining) or 10 mM sodium citrate/0.05% Tween 20 buffer (pH 6) in 121 °C autoclave (20 min for p-Tau staining). Immunohistochemical signals were developed using ImmPRESS™ anti-mouse IgG (peroxidase) polymer detection kit (Vector Laboratories, Burlingame, CA, USA) and diaminobenzidine (ImmPACT™ DAB peroxidase substrate, Vector Laboratories). The sections were counterstained with hematoxylin. Neocortex sections from AD were used as positive tissue controls. For negative reagent controls, the primary antibodies were omitted, as previously described [21].

On light microscopic examination, Aβ plaque pathology was considered present within a brain region when extracellular Aβ-immunoreactive plaques were observed regardless of their type (e.g., diffuse plaques, cored plaques) [69] or density (i.e., focal, widespread). The density of region-specific Aβ plaques was graded as 0 (absent), 1 (focal), or 2 (widespread), as described previously [70]. Neuronal p-Tau pathology was deemed present within a brain region when there were p-Tau-immunoreactive neuropil threads, pretangle neuronal soma, neurofibrillary tangles, or their combinations [69,70] based on criteria adapted from a BrainNet Europe Consortium study [71]. The density of p-Tau neuropil threads was graded as 0 (absent), 1 (barely present at 100× magnification), 2 (easily noted at 100× magnification), or 3 (notable with naked eye inspection).

When examining pathology across brain regions, cases with detectable Aβ plaque pathology in any brain region examined (frontal neocortex or basal temporal neocortex or hippocampus) were considered “positive” for any Aβ plaque pathology. Cases without Aβ plaque pathology in all three brain regions were considered “negative” for any Aβ plaque pathology.

### 2.4. Statistical Analyses

We examined differences in sample characteristics between the NNTC and ADRC cases and among the four groups of PWH, PWoH NC, PWoH MCI, and PWoH AD dementia using chi-square tests for categorical variables and analyses of variance (ANOVA) for continuous variables. Chi-square tests examined group differences in the rates of Aβ and p-Tau pathology positivity (pathology grade ≥ 1) within specific regions of interest (frontal neocortex, basal temporal neocortex, hippocampus, entorhinal [p-Tau only], and transentorhinal cortex [p-Tau only]) and across regions. We also examined group differences in the prevalence of more widespread Aβ and p-Tau pathology (pathology grade > 1). Lastly, we examined group differences in the prevalence of conjoint positivity for Aβ and p-Tau pathology in any brain region. Analyses comparing pathology burden between PWH and PWoH were unadjusted for relevant demographic and clinical variables for the following two reasons: (1) the purpose of these statistical comparisons was for the non-HIV groups to serve as a basis of comparison for the level of AD pathology in PWH, and (2) the statistical adjustment of demographics when comparing groups mismatched on these demographics is problematic and not advised [72].

Among PWH only, a series of analyses of covariance (ANCOVA) were conducted to examine the mean differences in domain-specific T-scores by overall and region-specific amyloid and p-Tau pathology positivity while adjusting for relevant covariates. Considered covariates included demographics (age, sex, race/ethnicity, years of education), *APOE*-ε4, and HIV disease variables (nadir CD4+ T-cell count, CD4+ T-cell count, plasma HIV-1 RNA load, estimated duration of HIV infection, and ART use), and clinical factors (major depressive disorder, alcohol use disorder, other substance use disorders, diabetes mellitus, hypertension and hyperlipidemia) from the last visit prior to death. Covariates that significantly related to the prevalence of any or widespread Aβ or p-Tau pathology in any brain region or the outcome of interest (domain-specific T-scores) at *p* ≤ 0.05 in univariate analyses were included in statistical models and were retained if significant in the multivariable model.

Secondarily, we examined the prevalence of AD pathology and the AD pathology and cognition association by sex; however, these analyses were hypotheses-generating due to the small number of women in our sample. For that same reason, effect sizes were reported and guided the interpretation of the results.

Analyses were performed using SPSS 26 (SPSS Inc., Chicago, IL, USA). Significance was defined as α = 0.05 (two-sided).

## 3. Results

See Table 1 for characteristics of the largest sample (those with Aβ and p-Tau pathology characterization in the frontal neocortex) by group.

**Figure 1:**
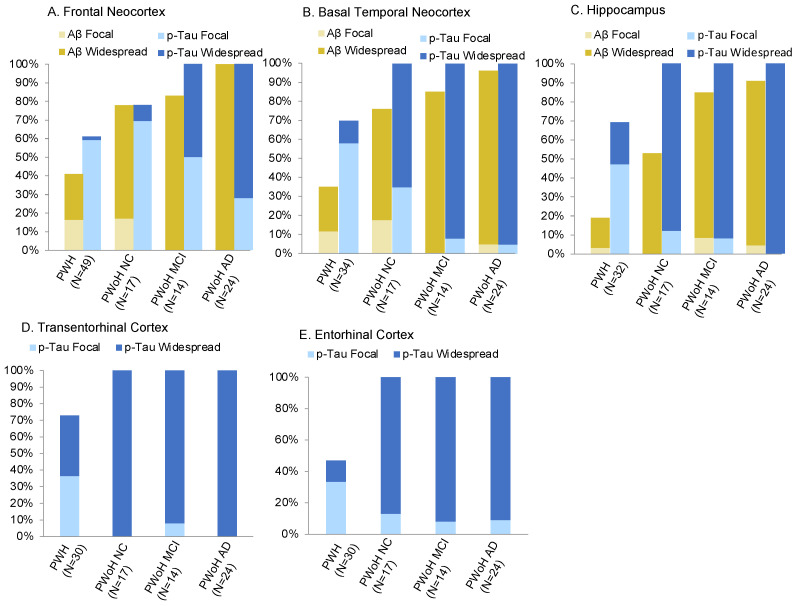
Region-specific prevalence of Aβ and p-Tau pathology in older PWH (NNTC cohort) and PWoH (ADRC cohort). NC = normal cognition. MCI = mild cognitive impairment. AD = Alzheimer’s disease dementia.

See Table 2 for sample size with region-specific pathological characterization data and the number of PWH and PWoH classified as pathology positive versus negative by brain region. A comparison of the proportion of region-specific Aβ and p-Tau positive cases among PWH and PWoH cases is presented in Figure 1.

### 3.1. Region-Specific Aβ Prevalence

Aβ positivity (grade > 0) ranged from 19% in the hippocampus to 41% in the frontal neocortex among PWH. The prevalence of more widespread Aβ pathology (grade > 1) among PWH ranged from 16% in the hippocampus to 24% in the frontal neocortex. Among NC PWoH, Aβ positivity (grade > 0) by brain region ranged from 53% in the hippocampus to 78% in the frontal neocortex. The prevalence of more widespread Aβ pathology (grade > 1) ranged from 53% in the hippocampus to 61% in the frontal neocortex. Among PWoH with MCI, Aβ positivity rates were similar across brain regions (85–86%), and, in almost all of these cases, the Aβ pathology was widespread. Lastly, among PWoH with AD dementia, Aβ positivity ranged from 92% in the hippocampus to 100% in the frontal neocortex, with widespread Aβ pathology found in almost all cases.

### 3.2. Region-Specific p-Tau Prevalence

P-Tau positivity (grade > 0) among PWH ranged from 47% in the entorhinal cortex to 73% in the transentorhinal cortex. The prevalence of more widespread p-Tau pathology (grade > 1) among PWH ranged from 2% in the frontal neocortex to 37% in the transentorhinal cortex. Among NC PWoH, p-Tau positivity ranged from 78% in the frontal neocortex to 100% in the basal temporal neocortex, transentorhinal cortex, entorhinal cortex, and hippocampus. The prevalence of more widespread p-Tau among NC PWoH ranged from 11% in the frontal neocortex to 100% in the transentorhinal cortex. In the PWoH with MCI group, 100% of cases were p-Tau positive in at least one brain region and more widespread p-Tau pathology (grade > 1) ranging from 54% in the frontal neocortex to 93% in the basal temporal neocortex, hippocampus, and entorhinal cortex. Lastly, among PWoH with AD dementia, 100% of cases were p-Tau positive in at-least one brain region, and more widespread p-Tau ranging from 71% in the frontal neocortex to 100% in the hippocampus and transentorhinal cortex.

### 3.3. Prevalence of Conjoint Aβ and p-Tau Positivity

Overall, 53% of PWH were Aβ and p-Tau positive in at least one brain region. Seventeen percent of PWH had more widespread Aβ and p-Tau pathology in at least one brain region. Aβ and p-Tau positivity was observed in at least one brain region in 82% of NC PWoH, 87% of PWoH with MCI, and 100% of PWoH with AD dementia. More widespread Aβ and p-Tau pathology was observed in at least one brain region in 65% of NC PWoH, 86% of PWoH with MCI, and 100% of PWoH with AD dementia.

### 3.4. Comparison of AD Pathology between PWH and PWoH

Generally speaking, Aβ and p-Tau positivity were significantly lower in PWH versus PWoH regardless of cognitive status of the PWoH (*p*s < 0.05). As expected, these differences were smallest when comparing PWH to NC PWoH and largest when comparing PWH to PWoH with AD dementia, although the prevalence of more widespread p-Tau pathology was at or near 100% in the medial temporal lobe regions of PWoH across the aging-MCI-AD spectrum. The majority of the Aβ positive cases across brain regions had widespread Aβ pathology in both PWH (60–83%) and PWoH (77–100%) groups. In contrast, among p-Tau positive cases across brain regions, the proportion of cases with widespread p-Tau cases was far lower in the PWH sample compared to all PWoH groups for all brain regions with the exception of PWH versus NC PWoH in the frontal neocortex. There were comparisons where PWH and PWoH did not significantly differ; prevalence rates of any p-Tau pathology (*X*^2^ = 1.60, *p* = 0.21) and widespread p-Tau pathology (*X*^2^ = 2.53, *p* = 0.11) in the frontal neocortex did not significantly differ between PWH and NC PWoH. Additionally, rates of Aβ positivity in *any* region did not significantly differ between PWH and NC PWoH (*X*^2^ = 2.89, *p* = 0.09) or PWoH with MCI (>0: *X*^2^ = 3.74, *p* = 0.05). Similarly, PWH did not significantly differ from any of the PWoH groups when examining rates of p-Tau positivity in *any* brain region (all *p*s > 0.44).

### 3.5. Relationship between AD Pathology and Antemortem Cognitive Function among PWH

In PWH only, we examined how AD pathology related to antemortem cognitive function while adjusting for relevant covariates. Among the considered covariates, *APOE*-ε4 status related to a higher likelihood of any Aβ pathology (*X*^2^ = 2.72, *p* = 0.09) and widespread Aβ pathology (*X*^2^ = 3.27, *p* = 0.07) at trend level. Higher age (F(1,34) = 3.45, *p* = 0.07), higher nadir CD4+ T-cell count (F(1,22), *p* = 0.03) and presence of hypertension (*X*^2^ = 7.01, *p* = 0.03) related to a higher likelihood of widespread Aβ pathology at, at least, trend level. Higher age related to a higher likelihood of widespread p-Tau pathology (F(1,29) = 4.28, *p* = 0.048). Greater years of education also related to a higher likelihood of widespread p-Tau pathology (F(1,28) = 5.78, *p* = 0.02), which was curious; however, a similar finding of greater education relating to higher likelihood of Aβ pathology was reported in our prior study in an NNTC postmortem cohort [57]. Hypertension significantly related to poorer learning (F(1,32) = 5.11, *p* = 0.03), memory (F(1,32) = 4.98, *p* = 0.03), and verbal fluency (F(1,31) = 4.52, *p* = 0.04) T-scores. Hyperlipidemia significantly related to poorer executive function T-scores (F(1,30) = 4.77, *p* = 0.04). *APOE*-ε4 status significantly related to poorer attention/working memory (F(1,42) = 4.28, *p* = 0.04), learning (F(1,43) = 7.74, *p* = 0.008), and memory (F(1,43) = 11.58, *p* = 0.001) T-scores. Due to their significant associations with either AD pathology or domain-specific T-scores, age, education, and *APOE*-ε4 status were included in statistical models and were retained if significant in the multivariable model at *p* ≤ 0.10. Because nadir CD4+ T-cell count was missing in 18 participants and hypertension and hyperlipidemia in 13 participants, follow-up sensitivity analyses were conducted that adjusted for nadir CD4, hypertension and hyperlipidemia to test for any substantive change in results.

Comparisons of the means and SDs of domain T-scores by pathology positivity status are displayed in Table 3 for memory-related domains and in Table 4 for other cognitive domains. Among PWH, p-Tau positivity status in any brain region did not relate to cognitive domain scores. Because 100% of cases were p-Tau positive in at-least one brain region, we examined cognitive domain scores by the positivity status of widespread p-Tau in any brain region and found no significant differences. There were no significant differences in cognitive domain scores by region-specific Aβ positivity status, although associations between Aβ positivity status in the frontal neocortex and poorer memory-related domain scores approached significance (*p* = 0.06–0.08). When examining Aβ positivity status in any brain region, there were significant differences specifically for the memory-related domains, whereby learning and recall domain scores were significantly lower for Aβ positive versus negative cases. When examining positive status for widespread Aβ pathology in any brain region, there were significant differences for the memory-related and verbal fluency domains, whereby learning, recall, and verbal fluency T-scores were significantly lower for those cases positive for widespread Aβ pathology in any region versus negative. There was a marginally significant difference in attention/working memory T-scores between those with versus without widespread Aβ pathology in any region with lower T-scores in those with widespread Aβ pathology (*p* = 0.05). See Table 5 for a summary of results regarding pathology relationships with cognitive function. Statistical results did not change substantively after adjusting for nadir CD4+ T-cell count and hypertension.

### 3.6. AD Pathology Prevalence in Women versus Men with HIV

Among PWH, women were significantly less likely to self-identify as White (*X*^2^ = 7.08, *p* = 0.008; Supplementary Table S1), more likely to self-identify as Hispanic (*X*^2^ = 3.69, *p* = 0.05), and found to have a lower mean WRAT-3 score (F(1,41) = 9.50, *p* = 0.004) compared to men. There were no additional statistical differences in demographic or clinical characteristics between the sexes. Supplementary Table S2 presents a comparison of the Aβ and p-tau pathology positivity rates between women and men with HIV. With the exception of frontal neocortex Aβ pathology, women had higher rates of Aβ and p-Tau positivity across brain regions, although these results were not statistically significant, likely due to the small sample of women. The phi coefficient (φ), an effect size measure for chi-square analyses (0.1 = small, 0.3 = medium, 0.5 = large) indicated that the largest sex differences in positivity rates were small-to-medium and medium sized for frontal neocortex p-Tau pathology (80% in women vs. 56.4% in men, φ = −0.20), hippocampal p-Tau pathology (85.7% in women vs. 64.0% in men, φ = −0.19), and basal temporal neocortex Aβ pathology (62.5% in women versus 26.9% in men, φ = −0.32).

### 3.7. AD Pathology and Cognitive Function Relationship in Women versus Men with HIV

#### 3.7.1. Women with HIV

Means and SDs of domain T-scores by pathology positivity status and ANOVA results for sex-stratified samples are displayed in Supplementary Table S3 for memory-related domains and in Supplementary Table S4 for other cognitive domains. In women, we found the presence of any Aβ pathology to be strongly associated with lower learning (*d* = 1.46) and recall (*d* = 1.49) T-scores, and these associations were even stronger when examining the presence of widespread Aβ pathology (learning: *d* = 2.06; recall: *d* = 2.74). The presence of any Aβ pathology also showed strong relationships with lower executive function (*d* = 0.70), attention/working memory (*d* = 0.92), and complex motor function (*d* = 0.90) T-scores. The presence of widespread Aβ pathology also showed strong relationships with lower executive function (*d* = 1.74), attention/working memory (*d* = 0.84), and verbal fluency (*d* = 0.93) T-scores. The presence of widespread p-Tau pathology showed strong relationships with poorer learning (*d* = 1.50) and recall (*d* = 1.39), speed of information processing (*d* = 0.79), and attention/working memory (*d* = 0.91) T-scores. (See Supplementary Table S5 for a summary of results regarding pathology relationships with cognitive function relationships in sex-stratified samples.)

#### 3.7.2. Men with HIV

In men, the presence of Aβ pathology in any brain region showed strong relationships with lower learning (*d* = 0.74), recall (*d* = 0.63), and verbal fluency (*d* = 0.71) T-scores scores and a moderate relationship with recall T-scores (*d* = 0.63) (Supplementary Tables S3 and S4). The presence of widespread Aβ pathology in any brain region showed strong relationships with lower recall (*d* = 0.79), verbal fluency (*d* = 0.87), and attention/working memory (*d* = 1.23) T-scores. Positivity for any or widespread p-Tau pathology did not relate to any domain T-score.

## 4. Discussion

As the lifespan of PWH approaches that of PWoH, there is growing interest in the prevalence and potential contribution of age-related neuropathology (e.g., Aβ, p-Tau) on neuropsychological phenotypes and AD risk among PWH. Studies have previously reported the presence of Aβand p-Tau pathology in the brain tissue of PWH; however, the regional specificity of this pathology, how pathology burden compares to that seen across the aging-MCI-AD dementia trajectory of PWoH and the clinical neurocognitive manifestation of this pathology are major knowledge gaps. Our study used the gold-standard method of assessing AD pathology burden (neuropathological characterization in brain tissue) in a sample limited to adults at-least 50 years of age (age range: 50–68) to address these questions. The strength of this approach is that initial AD pathogenesis typically occurs in the 5th and 6th decades of life and is often not detected by PET [73].

We found that, depending on region, the prevalence of any Aβ pathology ranged from 19% in the hippocampus (widespread in 16%) to 41% in the frontal neocortex (widespread in 24%) among PWH. The prevalence of any p-Tau pathology was at or near 70% (widespread in 10–30%) in all regions except the entorhinal cortex where the prevalence was 47%. Generally, Aβ and p-Tau pathology positivity rates were 30–50% lower in PWH versus NC PWoH across brain regions, except for similar rates of frontal neocortex p-Tau positivity in PWH and NC PWoH. Expectedly, the difference in pathology prevalence between PWH versus PWoH increased in magnitude with advancing stage of the aging-MCI-AD spectrum in PWoH, although widespread p-Tau pathology in the medial temporal lobe regions was prevalent in approximately 100% of PWoH regardless of cognitive status. Among PWH, Aβ positivity in any brain region related specifically to poorer memory performance, and this relationship was consistent across sexes. P-Tau positivity rates did not relate to cognitive performance in any domain in the overall sample; however, sex-stratified analyses revealed a strong, albeit non-significant, relationship of p-Tau positivity to poorer memory performance among women only.

Overall, our results indicate that AD pathology is present in a sizable portion of PWH, aged 50 years and older; however, the prevalence in PWH was considerably lower than those in samples of NC, MCI, and AD PWoH that were substantially older than the PWH. Our results do not provide support for accelerated aging or increased AD risk hypotheses in PWH; however, the results also do not refute the hypotheses either because the higher levels of AD pathology in PWoH are likely due to their older age (30-year mean difference). The rates of AD pathology among PWH reported in this study are similar to those reported in a prior post-mortem study by Morgello et al. (2021) [19] that examined AD pathology in the frontal and temporal lobe regions of 194 PWH and 63 PWoH from the Manhattan Brain Bank site of the NNTC. They reported Aβ pathology in 28% of PWH and p-Tau pathology in 60% of PWH; however, they did not differentiate between frontal and temporal regions and the sample was not limited to older PWH (age range: 21–86) [19]. Unlike the current study, PWH and PWoH groups in the Morgello et al. (2021) study were demographically matched, but they similarly found lower rates of Aβ pathology in PWH (rates of p-Tau pathology were similar between PWH and PWoH). The authors speculated that this could be due to a survivor effect in older PWH whereby advantageous health factors that allowed them to live longer with HIV may also mitigate risk of Aβ pathology. Conversely, some postmortem and neuroimaging studies with better age-matched samples of PWH and PWoH reported no difference in the prevalence of Aβ plaques between PWH (on ART) and PWoH [22,23,24,25,26], while other postmortem studies reported a higher prevalence of neocortical Aβ plaques in PWH versus PWoH [13,14,18,21]. In the Morgello et al. (2021) study, they found that longer HIV disease duration was a predictor of Aβ pathology of equal strength as chronological age [19], which suggests a contribution of HIV-related mechanisms to Aβ pathology. Overall, the literature regarding Aβ pathology remains equivocal possibly due to differences across studies in age of the study sample, levels of viral suppression and ART use, the brain regions examined, and the method of Aβ detection with PET tracers being less sensitive to the diffuse Aβ plaques commonly seen in PWH. The research regarding p-Tau in PWH is limited.

The presence of any or widespread p-Tau and Aβ pathology in the frontal neocortex showed the smallest difference between PWH and NC PWoH and, in fact, the p-Tau difference was non-significant despite the 30-year age difference. Moreover, the relationship between Aβ pathology prevalence and memory was driven by Aβ pathology in the frontal neocortex. These findings suggest an adverse or compounding effect of HIV- and aging-related overlapping mechanisms on neurodegenerative disease processes specifically in the frontal lobe that may include chronic inflammation, immune senescence, mitochondrial dysfunction, and oxidative stress [2,3,5,74,75]. This aligns with the literature in the pre- and post-ART eras showing that HIV has a particular affinity for the frontal-striatal circuits [76,77,78,79]. HIV-seropositivity is associated with grey matter atrophy [80,81], cortical thinning [82], synaptic damage [83], gliosis [22], and lower N-Acetyl-Aspartate [84] in the frontal lobe.

Aβ plaques and p-Tau lesions are the hallmarks of AD pathogenesis, but are also seen in normal aging and in other pathogenic processes, such as chronic traumatic encephalopathy (CTE) and substance use disorders [85,86]. Aβ and p-Tau pathology are common among cognitively normal older adults [87] and, in this group, a higher burden of Aβ and p-Tau is associated with a higher risk of future cognitive decline [87,88,89]. We probed the clinical significance of Aβ and p-Tau pathology in PWH by examining how its presence relates to antemortem cognitive function. The results partially supported our hypotheses with Aβ, but not p-Tau, pathology relating mostly to AD-sensitive domains in the overall sample of PWH. We found that Aβ positivity status in any brain region significantly related to poorer learning and recall scores. The presence of widespread Aβ pathology in any region significantly related to poorer verbal fluency performance in addition to learning and recall. In region-specific analyses, these associations were driven by frontal neocortex Aβ pathology. The frontal neocortex is one of the initial sites of Aβ plaque deposition in AD pathogenesis [9] and the domains of learning, recall, and verbal fluency are domains impacted in early-stage AD [90]. Thus, the specificity of the relationship to frontal neocortex Aβ pathology and to those particular domains suggests that the presence of Aβ pathology may be a harbinger of an AD-like neurodegenerative trajectory. These findings are supported by our prior findings that the presence of frontal neocortex Aβ pathology significantly related to an aMCI, but not to HIV-associated cognitive impairment, diagnosis among 74 middle aged to older post-mortem PWH [57]. Additionally, Soontornniyomkij et al., (2012) found a relationship between the presence of Aβ pathology and HAND but only among PWH with the *APOE*-ε4 allele [70].

Our clinicopathologic findings among PWH deviated from an AD-like profile in that p-Tau pathology was the least prevalent in the entorhinal cortex and the presence of widespread p-Tau pathology did not relate to cognitive function. Whereas the entorhinal cortex is an initial site of p-Tau pathogenesis in AD, we saw the lowest prevalence rate of p-Tau pathology in the entorhinal cortex among PWH. P-Tau pathology is a central pathological feature in a spectrum of conditions termed tauopathies including CTE and primary, and these conditions show differing patterns of regional p-Tau pathology distribution [91]. P-Tau pathology is closely tethered to cognitive symptoms in AD [92,93], but did not relate to cognitive performance in the PWH sample, although the sex-stratified analysis hinted at a female-specific relationship. The inconsistencies in the profile of p-Tau pathology in our PWH sample versus AD suggests that other etiologic origins to p-Tau pathology in HIV should be considered. Our findings align with a postmortem study of AD pathology characterization in 88 to 159 (brain region dependent) postmortem PWH (ages 26–70) by Soontornniyomkij et al. (2019) [20], although both studies were conducted in the NNTC cohort and so not independent of one another. Soontornniyomkij et al. found that prefrontal and putamen Aβ plaques relate to lower speed of information processing and attention/working memory, respectively; however, regional p-Tau lesions did not relate to any cognitive domains [20]. We can only speculate as to why this is the case, but evidence suggests that Aβ can interact with HIV-related disease mechanisms to become more neurotoxic. Tat protein, which is expressed in HIV-infected macrophages/microglia [94], is known to interact directly with Aβ and create a Aβ-Tat complex that is more neurotoxic than Aβ alone [95]. Furthermore, Aβ and Tat can synergistically potentiate the expression of inflammatory genes in human brain microvascular endothelial cells [96]. Therefore, it is possible that the functional significance of Aβ is amplified in the context of HIV infection.

Although the small number of women mandates caution in interpreting results in that subsample, the strong, female-specific relationship between p-Tau pathology and cognition is interesting and deserves further exploration. There are important sex differences in both AD and HAND with the prevalence higher in females than males for both conditions [39,40,49]. While it is unknown why a p-Tau pathology and cognition relationship may be female-specific in our sample, this finding aligns with the AD literature showing a stronger relationship between p-Tau burden and cognitive performance in women versus men in both a PET and a postmortem study [45,56]. Among PWH, Pulliam et al. (2019) found sex differences in the proteins extracted from plasma neuronal-derived exosomes that related to cognitive impairment with one of these proteins including total-Tau in women but not men [97]. This suggests mechanistic differences associated with cognitive impairment in PWH; however, there are also sociodemographic differences in women versus men with HIV in the U.S. that may contribute to cognitive reserve disparities. The greater prevalence of psychosocial risk factors (e.g., poverty, low education, substance use, depression, early life trauma, barriers to healthcare) in women versus men with HIV in the U.S. [98,99] are believed to result in a lower cognitive reserve in women with HIV making them more susceptible to cognitive deficits resulting from brain insult including HIV infection and neurodegenerative pathology [100,101]. Indeed, mean WRAT-3 Reading scores, a common proxy for cognitive reserve, were significantly lower in women versus men with HIV in the current sample. In support of cognitive reserve disparities underlying sex differences in HAND, an earlier study from our group found that the significantly higher prevalence of HAND in women versus men with HIV was eliminated when adjusting for scores on the WRAT-3 Reading subtest, a common proxy for cognitive reserve [102]. Psychosocial risk factors are also associated with higher levels of stress and inflammation, which also play a role in risk for HAND and AD [103,104,105,106,107]. We believe that our findings will serve as a springboard for future studies designed to examine the influence of sex on links between neuropathology and cognition among PWH.

Major strengths include our examination of AD pathology by brain region and its relationship to antemortem, domain-specific cognitive performance. Additionally, this represents the first study to compare AD pathology rates in PWH to a cohort of PWoH that spanned the health aging-MCI-AD spectrum and to conduct sex comparisons of AD pathology and its cognitive correlates among PWH. Our study has limitations. The age difference between PWH and PWoH cohorts was large with the PWoH cohort being considerably older, which limits our ability to assess the relationship between HIV serostatus and AD pathology prevalence. We did not apply a multiple comparison correction because we had specific, a priori hypotheses about which cognitive domains would relate to pathology; however, an inflated type I error should be considered when interpreting results. As this was an autopsy cohort, it was characterized by advanced medical morbidity, which limits the generalizability to the general population of PWH. The generalizability of our results is also limited in that our sample was predominantly male and white. Thus, we are cautious in interpreting results from the sex-stratified samples and view those findings as hypothesis-generating. A strength of our study was that we conducted neuropathological characterization in multiple brain regions; however, these were limited to AD-relevant brain regions selected a priori and cannot be generalized to other brain regions. Lastly, the cross-sectional nature of neuropathological characterization precludes any investigations or interpretations of the temporal direction of the pathology and cognition relationship or the stability of this relationship over time.

## 5. Conclusions

Our results indicate that Aβ and p-Tau pathology are present in AD-relevant regions in a sizable portion of mid-to-late life PWH; however, with the exception of p-Tau pathology in the frontal neocortex, the severity of pathology is considerably less (30–50%) than in older, NC PWoH. The prevalence of Aβ and p-Tau pathology among PWH indicates the importance of utilizing multiple AD-associated clinical and biological markers in order to differentiate healthy aging from an AD trajectory among PWH. Studies with better age-matched PWoH are more well suited to examine the effect of HIV status on Aβ and p-Tau pathology. Among PWH, the presence of frontal neocortex Aβ pathology significantly predicted poorer performance in the AD-sensitive domains of learning, recall, and verbal fluency performance; however, the lack of a relationship between p-Tau pathology and cognitive performance in the overall sample may suggest etiologies other than AD. An interesting female-specific relationship between p-Tau pathology and cognition necessitates further exploration.

## Figures and Tables

**Table 1:** Sample characteristics by group.

	PWH (NNTC Cases, *n* = 49)	PWoH (ADRC Cases)			Test Statistic, *p*-Value
		**NC (*n* = 20)**	**MCI (*n* = 15)**	**AD (*n* = 25)**	
**Demographics**					
Age, mean (SD)	57.37 (5.04)	89.10 (6.95)	89.33 (6.85)	88.04 (7.44)	F (3,105) = 225.17, *p* < 0.001
Education years, mean (SD)	12.69 (2.91)	14.90 (2.81)	14.93 (2.71)	15.08 (3.46)	F (3,105) = 5.00, *p* < 0.001
Sex, *n* (% male)	39 (79.59%)	7 (35.00%)	8 (53.33%)	13 (52.00%)	*X*^2^ = 14.08, *p* = 0.003
Race/ethnicity					
White, *n* (%)	28 (57.14%)	18 (90.00%)	15 (100.00%)	25 (100.00%)	*X*^2^ = 26.11, *p* < 0.001
Black, *n* (%)	15 (30.61%)	0 (0.00%)	0 (0.00%)	0 (0.00%)	*X*^2^ = 21.30, *p* < 0.001
Hispanic, *n* (%)	6 (12.24%)	1 (5.00%)	0 (0.00%)	0 (0.00%)	*X*^2^ = 5.58, *p* = 0.13
Other, *n* (%)	0 (0.00%)	1 (5.00%)	0 (0.00%)	0 (0.00%)	*X*^2^ = 4.49, *p* = 0.21
*APOE* ε4 carrier, *n* (%)	11 (22.92%)	4 (21.05%)	5 (33.33%)	13 (52.00%)	*X*^2^ = 7.56, *p* = 0.06
WRAT-3 reading subtest, mean (SD)	47.7 (9.6)	NA	NA	NA	
**Antemortem Clinical Comorbidities**					
History of alcohol use disorder, *n* (%)	28 (60.87%)	0 (0.00%)	1 (11.11%)	1 (8.33%)	*X*^2^ = 24.82 *p* < 0.001
History of substance use disorder, *n* (%)	33 (71.74%)	0 (0.00%)	0 (0.00%)	0 (0.00%)	*X*^2^ = 40.66 *p* < 0.001
Major depressive disorder, *n* (%)	32 (69.57%)	1 (14.29%)	3 (30.00%)	3 (21.43%)	*X*^2^ = 17.62 *p* < 0.001
Hypertension, *n* (%)	12 (33.33%)	4 (36.36%)	7 (87.50%)	6 (60.00%)	*X*^2^ = 8.69, *p* = 0.03
Diabetes, *n* (%)	5 (13.89%)	2 (10.53%)	1 (8.33%)	3 (17.65%)	*X*^2^ = 1.91, *p* = 0.59
Hypercholesterolemia, *n* (%)	9 (25.00%)	5 (41.67%)	8 (88.89%)	9 (75.00%)	*X*^2^ = 16.02, *p* = 0.001
**HIV Disease Characteristics**					
Nadir CD4+ T-cell count (cells/μL), mean (SD)	97.87 (138.56)	NA	NA	NA	NA
Antemortem CD4+ T-cell count (cells/μL), mean (SD)	198.73 (233.76)	NA	NA	NA	NA
Antemortem detectable plasma HIV-1 RNA load (≥50 copies/mL), *n* (%)	32 (65.31%)	NA	NA	NA	NA
Duration of HIV disease, years, mean (SD)	14.75 (6.71)	NA	NA	NA	NA
Antemortem ART, *n* (% prescribed)	34 (85%)	NA	NA	NA	NA

Note. PWH = people with HIV. PWoH = people without HIV. NC = normal cognition. MCI = mild cognitive impairment. AD = Alzheimer’s disease dementia. ART = antiretroviral therapy. *APOE* ε4 = apolipoprotein E ε4 allele. WRAT-3 = Wide Range Achievement Test-version 3. Differences in sample characteristics by NNTC/ADRC group were tested using chi-square tests for categorical variables and univariate ANOVAs for normally-distributed continuous variables. NA: not available.

**Table 2:** Number of PWH and PWoH classified as pathology positive (Path+) versus negative (Path−) by brain region.

	PWH (NNTC Cohort)		PWoH NC		PWoH MCI		PWoH AD	
	Path+	Path−	Path+	Path−	Path+	Path−	Path+	Path−
**Aβ**								
Frontal neocortex	20	29	14	3	12	2	24	0
Basal temporal neocortex	12	22	13	4	12	2	24	0
Hippocampus	6	26	9	7	12	2	23	1
Any brain region	23	16	14	3	12	2	24	0
Widespread Aβ (any brain region)	12	24	11	6	12	2	24	0
**p-Tau**								
Frontal neocortex	30	19	14	3	14	0	24	0
Basal temporal neocortex	24	10	17	0	14	0	24	0
Trans-entorhinal cortex	22	8	17	0	14	0	24	0
Entorhinal cortex	14	16	17	0	14	0	24	0
Hippocampus	22	10	17	0	14	0	24	0
Any brain region	41	1	17	0	14	0	24	0
Widespread p-Tau (any brain region)	15	16	17	0	13	1	24	0

Note. PWH = people with HIV. PWoH = people without HIV. NC = normal cognition. MCI = mild cognitive impairment. AD = Alzheimer’s disease dementia. Path+ = pathology positive. Path− = pathology negative. Pathology positive was defined as pathology grade ≥ 1 with the density of Aβ plaques graded as 0 (absent), 1 (focal), or 2 (widespread), and the density of p-Tau neuropil threads graded as 0 (absent), 1 (barely present at 100× magnification), 2 (easily noted at 100× magnification), or 3 (notable with naked eye inspection).

**Table 3:** Comparison of memory performance (demographically-adjusted T-scores) between PWH with (Path+) versus without (Path−) Aβ and p-Tau pathology.

	Learning			Recall		
	Path+ Mean (SD)	Path− Mean (SD)	ANOVA Results	Path+ Mean (SD)	Path− Mean (SD)	ANOVA Results
**Aβ**						
Frontal neocortex	38.3 (7.4)	43.2 (9.8)	F(1,43) = 3.2. *p* = 0.08	37.6 (9.3)	43.0 (8.9)	F(1,43) = 3.8, *p* = 0.06
Basal temporal neocortex	39.3 (8.4)	44.6 (9.8)	F(1,31) = 2.3, *p* = 0.14	38.9 (8.6)	43.9 (9.9)	F(1,31) = 2.0, *p* = 0.16
Hippocampus	42.3 (7.4)	42.2 (10.5)	F(1,29) = 0.001, *p* = 0.98	42.3 (7.4)	42.2 (10.5)	F(1,29) = 0.001, *p* = 0.98
Any brain region	39.1 (7.1)	46.5 (10.5)	F(1,35) = 6.6, *p* = 0.01	38.7 (9.0)	45.2 (9.7)	F(1,35) = 4.5, *p* = 0.04
Widespread Aβ any brain region)	37.5 (8.6)	44.9 (9.1)	F(1,32) = 4.8, *p* = 0.04	35.8 (8.4)	44.2 (9.2)	F(1,32) = 6.1, *p* = 0.02
**p-Tau**						
Frontal neocortex	42.4 (9.6)	39.2 (8.2)	F(1,43) = 0.3, *p* = 0.27	42.2 (8.8)	38.2 (10.0)	F(1,43) = 1.9, *p* = 0.17
Basal temporal neocortex	42.5 (10.9)	9, 43.6 (4.7)	F(1,31) = 0.08, *p* = 0.77	41.5 (10.2)	44.3 (8.0)	F(1,31) = 0.6, *p* = 0.46
Transentorhinal cortex	44.4 (9.9)	40.1 (10.5)	F(1,27) = 0.99, *p* = 0.33	43.4 (10.4)	40.7 (9.3)	F(1,27) = 0.4, *p* = 0.54
Entorhinal cortex	44.0 (7.7)	42.8 (12.1)	F(1,27) = 0.1, *p* = 0.75	44.0 (9.5)	41.7 (10.7)	F(1,27) = 0.4, *p* = 0.55
Hippocampus	44.6 (10.1)	39.5 (8.8)	F(1,29) = 1.9, *p* = 0.18	43.2 (10.2)	40.1 (9.6)	F(1,29) = 0.6, *p* = 0.43
Any brain region ^¥^	NA	NA	NA	NA	NA	NA
Widespread p-Tau (any brain region)	40.8 (7.9)	45.3 (11.6)	F(1,28), *p* = 0.22	40.5 (9.4)	44.3 (10.7)	F(1,28) = 1.1, *p* = 0.31

Note. Path+ = pathology positive. Path− = pathology negative. Means and standard deviations (SD) are from domain-specific, demographically-adjusted T-scores. Pathology positive was defined as pathology grade ≥ 1 with the density of Aβ plaques graded as 0 (absent), 1 (focal), or 2 (widespread), and the density of p-Tau neuropil threads graded as 0 (absent), 1 (barely present at 100× magnification), 2 (easily noted at 100× magnification), or 3 (notable with naked eye inspection). ^¥^ Comparison is not possible as 100% of cases were p-Tau positive in at-least one brain region. NA: not available.

**Table 4:** Comparison of performance (demographically-adjusted T-scores) in non-memory cognitive domains between PWH cases with (path+) versus without (path−) Aβ and p-Tau pathology.

	Executive Function			Speed of Information Processing			Attention/Working Memory			Verbal Fluency			Motor		
	Path+ Mean (SD)	Path− Mean (SD)	ANOVA Results	Path+ Mean (SD)	Path− Mean (SD)	ANOVA Results	Path+ Mean (SD)	Path−Mean (SD)	ANOVA Results	Path+ Mean (SD)	Path− Mean (SD)	ANOVA Results	Path+ Mean (SD)	Path− Mean (SD)	ANOVA Results
**Aβ**															
Frontal neocortex	53.7 (13.2)	51.1 (11.1)	F(1,41) = 0.5, *p* = 0.49	43.6 (10.7)	46.1 (11.0)	F(1,40) = 0.5, *p* = 0.48	45.1 (8.4)	49.0 (9.8)	F(1,42) = 1.9, *p* = 0.18	45.6 (8.4)	50.6 (12.7)	F(1,42) = 2.1, *p* = 0.15	38.2 (9.5)	39.8 (9.8)	F(1,42) = 0.2, *p* = 0.62
Basal temporal neocortex	51.6 (15.1)	54.6 (11.7)	F(1,31) = 0.4, *p* = 0.54	46.7 (6.4)	49.0 (11.3)	F(1,29) = 0.4, *p* = 0.55	45.7 (11.0)	48.1 (7.1)	F(1,31) = 0.6, *p* = 0.46	46.8 (10.0)	53.0 (12.1)	F(1,31) = 2.1, *p* = 0.16	41.3 (9.0)	41.5 (8.8)	F(1,31) = 0.01, *p* = 0.93
Hippo-campus	49.3 (3.7)	54.7 (14.1)	F(1,29) = 0.7, *p* = 0.41	49.2 (7.2)	48.7 (10.7)	F(1,27) = 0.01, *p* = 0.92	41.9 (5.8)	48.3 (8.9)	F(1,29) = 2.4, *p* = 0.13	46.8 (10.5)	52.2 (12.2)	F(1,29) = 0.8, *p* = 0.36	41.3 (12.6)	41.3 (8.3)	F(1,29) = 0.0, *p* = 1.0
Any brain region	21, 53.8 (12.5)	16, 53.4 (12.1)	F(1,35) = 0.01, *p* = 0.92	20, 44.7 (10.3)	15, 49.1 (11.8)	F(1,33) = 1.4, *p* = 0.25	21, 46.5 (9.4)	16, 48.2 (7.0)	F(1,35) = 0.4, *p* = 0.55	21, 47.4 (9.0)	16, 54.2 (12.8)	F(1,35) = 3.5, *p* = 0.07	21, 39.0 (9.1)	16, 41.8 (9.7)	F(1,35) = 0.7, *p* = 0.40
Widespread Aβ any brain region)	10, 52.4 (14.8)	24, 53.9 (11.9)	F(1,32) = 0.1, *p* = 0.76	9, 45.1 (7.5)	23, 49.1 (10.8)	F(1,30) = 1.0, *p* = 0.32	10, 42.9 (8.0)	24, 49.1 (8.0)	F(1,32) = 4.2, *p* = 0.05	10, 44.5 (9.0)	24, 53.3 (11.7)	F(1,32) = 4.5, *p* = 0.04	10, 40.0 (10.1)	24, 41.1 (8.4)	F(1,32) = 0.2, *p* = 0.67
**p-Tau**															
Frontal neocortex	51.0 (11.4)	54.1 (13.1)	F(1,41) = 0.7, *p* = 0.42	46.9 (10.0)	41.4 (11.8)	F(1,40) = 2.5, *p* = 0.12	48.6 (8.2)	45.1 (11.0)	F(1,42) = 1.4, *p* = 0.24	48.8 (10.0)	48.1 (13.5)	F(1,42) = 0.03, *p* = 0.85	40.0 (9.0)	37.6 (10.7)	F(1,42) = 0.5, *p* = 0.46
Basal temporal neocortex	54.7 (13.7)	50.7 (10.0)	F(1,31) = 0.6, *p* = 0.43	48.1 (10.0)	48.7 (10.2)	F(1,29) = 0.02, *p* = 0.88	47.1 (8.9)	47.7 (7.8)	F(1,31) = 0.03, *p* = 0.87	49.6 (10.7)	54.4 (14.0)	F(1,31) = 1.1, *p* = 0.30	41.0 (8.8)	42.4 (8.8)	F(1,31) = 0.1, *p* = 0.71
Trans-entorhinal cortex	55.7 (14.2)	46.9 (6.8)	F(1,27) = 2.5, *p* = 0.13	50.8 (9.4)	45.1 (11.6)	F(1,25) = 1.7, *p* = 0.21	48.0 (9.0)	46.3 (8.1)	F(1,27) = 0.2, *p* = 0.66	52.9 (9.6)	49.1 (17.5)	F(1,27) = 0.5, *p* = 0.48	42.0 (8.3)	41.9 (9.5)	F(1,27) = 0.001, *p* = 0.97
Entorhinal cortex	57.2 (15.8)	50.2 (9.6)	F(1,27) = 2.1, *p* = 0.16	51.7 (9.5)	47.5 (10.6)	F(1,25) = 1.1, *p* = 0.30	46.0 (8.6)	49.1 (8.8)	F(1,27) = 0.9, *p* = 0.35	49.2 (8.8)	54.5 (13.8)	F(1,27) = 1.5, *p* = 0.23	43.4 (8.0)	41.0 (8.9)	F(1,27) = 0.5, *p* = 0.47
Hippo-campus	55.8 (14.1)	49.7 (10.1)	F(1,29) = 1.5, *p* = 0.23	49.7 (9.4)	47.0, (11.6)	F(1,27) = 0.4, *p* = 0.52	46.3 (7.6)	49.3 (10.9)	F(1,29) = 0.8, *p* = 0.37	53.0 (10.9)	47.8 (13.9)	F(1,29) = 1.3, *p* = 0.26	43.1 (8.3)	40.0 (9.4)	F(1,29) = 2.3, *p* = 0.14
Any brain Region ^¥^	NA	NA	NA	NA	NA	NA	NA	NA	NA	NA	NA	NA	NA	NA	NA
Widespread p-Tau (any brain region)	51.6 (11.1)	54.9 (15.1)	F(1,28) = 0.4, *p* = 0.51	48.6 (8.3)	49.6 (11.9)	F(1,26) = 0.1, *p* = 0.79	48.7 (8.1)	46.9 (9.3)	F(1,28) = 0.3, *p* = 0.58	51.3 (7.8)	51.7 (15.1)	F(1,28) = 0.01, *p* = 0.93			

Note. Path+ = pathology positive. Path− = pathology negative. Means and standard deviations (SD) are from domain-specific, demographically-adjusted T-scores. Pathology positive was defined as pathology grade ≥ 1 with the density of Aβ plaques graded as 0 (absent), 1 (focal), or 2 (widespread), and the density of p-Tau neuropil threads graded as 0 (absent), 1 (barely present at 100× magnification), 2 (easily noted at 100× magnification), or 3 (notable with naked eye inspection). ^¥^ Comparison is not possible as 100% of cases were p-Tau positive in at-least one brain region. NA: not available.

**Table 5:** Summary of results (statistical significance) examining relationships between Aβ and p-Tau positivity status and domain-specific cognitive function.

	Brain Region-Specific Pathology Positivity Status								Pathology Positivity Status in Any Brain Region			
Cognitive Domain	Frontal Neocortex		Basal Temporal Neocortex		Hippocampus		Trans-Entorhinal Cortex p-Tau	Entorhinal Cortex p-Tau	Any Aβ Pathology	Wide-Spread Aβ Pathology	p-Tau Pathology (Any Brain Region)	Wide-Spread p-Tau Pathology
	**Aβ**	**p-Tau**	**Aβ**	**p-Tau**	**Aβ**	**p-Tau**						
Learning	T	-	-	-	-	-	-	-	+	+	-	-
Recall	T	-	-	-	-	-	-	-	+	+	-	-
Executive Function	-	-	-	-	-	-	-	-			-	-
Speed of Information Processing	-	-	-	-	-	-	-	-	-	-	-	-
Attention/Working Memory	-	-	-	-	-	-	-	-	-	T	-	-
Verbal Fluency	-	-	-	-	-	-	-	-	T	+	-	-
Motor	-	-	-	-	-	-	-	-	-	-	-	-

Note. + indicates a significant association at *p* < 0.05. T indicates a relationship that is a statistical trend, *p* ≤ 0.10—indicates a relationship that is not significant or a trend.

## Data Availability

Biospecimens and clinical data collected as part of the NNTC and CHARTER protocols are available from the NNTC by request. Request number for data in present study was R545. Selected clinical data variables are available through the NNTC and CHARTER Query Tools to help requestors shape data and tissue requests and identify subpopulations of interest. Additional clinical data, beyond what is available from the Query Tools, is available from the NNTC and CHARTER cohorts and can be requested from the NNTC using the Data Request Application. Specimens or data obtained from the NNTC cannot be distributed to third-party companies or institutions without prior consent from the NNTC Steering Committee.

## Supplementary Materials

The following supporting information can be downloaded at: https://www.mdpi.com/article/10.3390/v15061319/s1. Table S1: Comparison of characteristics of PWH sample by sex, Table S2: Comparison of the Alzheimer’s disease pathology positivity rates between women and men with HIV, Table S3: Comparison of memory performance (demographically-adjusted T-scores) between pathology positive (+) versus pathology negative (−) PWH in sex-stratified samples. Table S4: Comparison of performance in non-memory cognitive domains (demographically-adjusted domain T-scores) between pathology positive (+) versus pathology negative (−) PWH in sex-stratified samples. Table S5: Summary of sex-stratified results examining relationships between Aβ and p-Tau positivity status and domain-specific cognitive function.

## References

[1] HIV Surveillance Report, 2020; Vol. 33. Published May 2022. http://www.cdc.gov/hiv/library/reports/hiv-surveillance.html.

[2] Alisky J.M. (2007). The Coming Problem of HIV-Associated Alzheimer’s Disease. Med. Hypoth..

[3] Cohen R.A., Seider T.R., Navia B. (2015). HIV Effects on Age-Associated Neurocognitive Dysfunction: Premature Cognitive Aging or Neurodegenerative Disease?. Alzheimer’s Res. Ther..

[4] Cruse B., Cysique L.A., Markus R., Brew B.J. (2012). Cerebrovascular Disease in HIV-Infected Individuals in the Era of Highly Active Antiretroviral Therapy. J. Neurovirol..

[5] Deeks S.G. (2011). HIV Infection, Inflammation, Immunosenescence, and Aging. Annu. Rev. Med..

[6] Stoff D.M., Goodkin K., Jeste D., Marquine M. (2017). Redefining Aging in HIV Infection Using Phenotypes. Curr. HIV/AIDS Rep..

[7] Horvath S., Levine A.J. (2015). HIV-1 Infection Accelerates Age According to the Epigenetic Clock. J. Infect. Dis..

[8] Durand M., Chartrand-Lefebvre C., Baril J.-G., Trottier S., Trottier B., Harris M., Walmsley S., Conway B., Wong A., Routy J.-P. (2017). The Canadian HIV and Aging Cohort Study—Determinants of Increased Risk of Cardio-Vascular Diseases in HIV-Infected Individuals: Rationale and Study Protocol. BMC Infect. Dis..

[9] Braak H., Braak E. (1991). Neuropathological Stageing of Alzheimer-Related Changes. Acta Neuropathol..

[10] Braak H., Thal D.R., Ghebremedhin E., Del Tredici K. (2011). Stages of the Pathologic Process in Alzheimer Disease: Age Categories from 1 to 100 Years. J. Neuropathol. Exp. Neurol..

[11] Bateman R.J., Xiong C., Benzinger T.L.S., Fagan A.M., Goate A., Fox N.C., Marcus D.S., Cairns N.J., Xie X., Blazey T.M. (2012). Clinical and Biomarker Changes in Dominantly Inherited Alzheimer’s Disease. N. Engl. J. Med..

[12] Morris J.C., Price J.L. (2001). Pathologic Correlates of Nondemented Aging, Mild Cognitive Impairment, and Early-Stage Alzheimer’s Disease. J. Mol. Neurosci..

[13] Achim C.L., Adame A., Dumaop W., Everall I.P., Masliah E. (2009). Neurobehavioral Research Center Increased Accumulation of Intraneuronal Amyloid Beta in HIV-Infected Patients. J. Neuroimmune Pharmacol..

[14] Esiri M.M., Biddolph S.C., Morris C.S. (1998). Prevalence of Alzheimer Plaques in AIDS. J. Neurol. Neurosurg. Psychiatry.

[15] Gelman B.B., Schuenke K. (2004). Brain Aging in Acquired Immunodeficiency Syndrome: Increased Ubiquitin-Protein Conjugate Is Correlated with Decreased Synaptic Protein but Not Amyloid Plaque Accumulation. J. Neurovirol..

[16] Green D.A., Masliah E., Vinters H.V., Beizai P., Moore D.J., Achim C.L. (2005). Brain Deposition of Beta-Amyloid Is a Common Pathologic Feature in HIV Positive Patients. AIDS.

[17] Rempel H.C., Pulliam L. (2005). HIV-1 Tat Inhibits Neprilysin and Elevates Amyloid Beta. AIDS.

[18] Smith D.B., Simmonds P., Bell J.E. (2014). Brain Viral Burden, Neuroinflammation and Neurodegeneration in HAART-Treated HIV Positive Injecting Drug Users. J. Neurovirol..

[19] Morgello S., Cortes E.P., Gensler G., Meloni G., Jacobs M.M., Murray J., Borukov V., Crary J.F. (2021). HIV Disease Duration, but Not Active Brain Infection, Predicts Cortical Amyloid Beta Deposition. AIDS.

[20] Soontornniyomkij V., Moore D.J., Gouaux B., Soontornniyomkij B., Sinsheimer J.S., Levine A.J. (2019). Associations of Regional Amyloid-β Plaque and Phospho-Tau Pathology with Biological Factors and Neuropsychological Functioning among HIV-Infected Adults. J. Neurovirol..

[21] Umlauf A., Soontornniyomkij B., Sundermann E.E., Gouaux B., Ellis R.J., Levine A.J., Moore D.J., Soontornniyomkij V. (2019). Risk of Developing Cerebral β-Amyloid Plaques with Posttranslational Modification among HIV-Infected Adults. AIDS.

[22] Solomon I.H., De Girolami U., Chettimada S., Misra V., Singer E.J., Gabuzda D. (2017). Brain and Liver Pathology, Amyloid Deposition, and Interferon Responses among Older HIV-Positive Patients in the Late HAART Era. BMC Infect. Dis..

[23] Murray J., Meloni G., Cortes E.P., KimSilva A., Jacobs M., Ramkissoon A., Crary J.F., Morgello S. (2022). Frontal Lobe Microglia, Neurodegenerative Protein Accumulation, and Cognitive Function in People with HIV. Acta Neuropathol. Commun..

[24] Ances B.M., Christensen J.J., Teshome M., Taylor J., Xiong C., Aldea P., Fagan A.M., Holtzman D.M., Morris J.C., Mintun M.A. (2010). Cognitively Unimpaired HIV-Positive Subjects Do Not Have Increased 11C-PiB: A Case-Control Study. Neurology.

[25] Vera J.H., Eftychiou N., Schuerer M., Rullmann M., Barthel H., Sabri O., Gisslen M., Zetterberg H., Blennow K., O’Brien C. (2021). Clinical Utility of β-Amyloid PET Imaging in People Living with HIV with Cognitive Symptoms. J. Acquir. Immune Defic. Syndr..

[26] Howdle G.C., Quidé Y., Kassem M.S., Johnson K., Rae C.D., Brew B.J., Cysique L.A. (2020). Brain Amyloid in Virally Suppressed HIV-Associated Neurocognitive Disorder. Neurol. Neuroimmunol. Neuroinflamm..

[27] Brew B.J., Crowe S.M., Landay A., Cysique L.A., Guillemin G. (2009). Neurodegeneration and Ageing in the HAART Era. J. Neuroimmune Pharmacol..

[28] Brew B.J., Letendre S.L. (2008). Biomarkers of HIV Related Central Nervous System Disease. Int. Rev. Psychiatry.

[29] Everall I., Vaida F., Khanlou N., Lazzaretto D., Achim C., Letendre S., Moore D., Ellis R., Cherner M., Gelman B. (2009). Cliniconeuropathologic Correlates of Human Immunodeficiency Virus in the Era of Antiretroviral Therapy. J. Neurovirol..

[30] Patrick C., Crews L., Desplats P., Dumaop W., Rockenstein E., Achim C.L., Everall I.P., Masliah E. (2011). Increased CDK5 Expression in HIV Encephalitis Contributes to Neurodegeneration via Tau Phosphorylation and Is Reversed with Roscovitine. Am. J. Pathol..

[31] Anthony I.C., Ramage S.N., Carnie F.W., Simmonds P., Bell J.E. (2006). Accelerated Tau Deposition in the Brains of Individuals Infected with Human Immunodeficiency Virus-1 before and after the Advent of Highly Active Anti-Retroviral Therapy. Acta Neuropathol..

[32] Fields J.A., Swinton M.K., Soontornniyomkij B., Carson A., Achim C.L. (2020). Beta Amyloid Levels in CSF of HIV-Infected People Vary by Exposure to Antiretroviral Therapy. AIDS.

[33] Clifford D.B., Fagan A.M., Holtzman D.M., Morris J.C., Teshome M., Shah A.R., Kauwe J.S.K. (2009). CSF Biomarkers of Alzheimer Disease in HIV-Associated Neurologic Disease. Neurology.

[34] Gisslén M., Krut J., Andreasson U., Blennow K., Cinque P., Brew B.J., Spudich S., Hagberg L., Rosengren L., Price R.W. (2009). Amyloid and Tau Cerebrospinal Fluid Biomarkers in HIV Infection. BMC Neurol..

[35] Jessen Krut J., Mellberg T., Price R.W., Hagberg L., Fuchs D., Rosengren L., Nilsson S., Zetterberg H., Gisslén M. (2014). Biomarker Evidence of Axonal Injury in Neuroasymptomatic HIV-1 Patients. PLoS ONE.

[36] Peterson J., Gisslen M., Zetterberg H., Fuchs D., Shacklett B.L., Hagberg L., Yiannoutsos C.T., Spudich S.S., Price R.W. (2014). Cerebrospinal Fluid (CSF) Neuronal Biomarkers across the Spectrum of HIV Infection: Hierarchy of Injury and Detection. PLoS ONE.

[37] Brew B.J., Pemberton L., Blennow K., Wallin A., Hagberg L. (2005). CSF Amyloid Beta42 and Tau Levels Correlate with AIDS Dementia Complex. Neurology.

[38] Cooley S.A., Strain J.F., Beaumont H., Boerwinkle A.H., Doyle J., Morris J.C., Benzinger T.L., Ances B.M. (2019). Tau Positron Emission Tomography Binding Is Not Elevated in HIV-Infected Individuals. J. Infect. Dis..

[39] Dreyer A.J., Munsami A., Williams T., Andersen L.S., Nightingale S., Gouse H., Joska J., Thomas K.G.F. (2022). Cognitive Differences between Men and Women with HIV: A Systematic Review and Meta-Analysis. Arch. Clin. Neuropsychol..

[40] Rubin L.H., Neigh G.N., Sundermann E.E., Xu Y., Scully E.P., Maki P.M. (2019). Sex Differences in Neurocognitive Function in Adults with HIV: Patterns, Predictors, and Mechanisms. Curr. Psychiatry Rep..

[41] Buckley R.F., Mormino E.C., Rabin J.S., Hohman T.J., Landau S., Hanseeuw B.J., Jacobs H.I.L., Papp K.V., Amariglio R.E., Properzi M.J. (2019). Sex Differences in the Association of Global Amyloid and Regional Tau Deposition Measured by Positron Emission Tomography in Clinically Normal Older Adults. JAMA Neurol..

[42] Hohman T.J., Dumitrescu L., Barnes L.L., Thambisetty M., Beecham G., Kunkle B., Gifford K.A., Bush W.S., Chibnik L.B., Mukherjee S. (2018). Sex-Specific Association of Apolipoprotein e with Cerebrospinal Fluid Levels of Tau. JAMA Neurol..

[43] Altmann A., Tian L., Henderson V.W., Greicius M.D. (2014). Sex Modifies the APOE-Related Risk of Developing Alzheimer Disease. Ann. Neurol..

[44] Oveisgharan S., Arvanitakis Z., Yu L., Farfel J., Schneider J.A., Bennett D.A. (2018). Sex Differences in Alzheimer’s Disease and Common Neuropathologies of Aging. Acta Neuropathol..

[45] Buckley R.F., Scott M.R., Jacobs H.I.L., Schultz A.P., Properzi M.J., Amariglio R.E., Hohman T.J., Mayblyum D.V., Rubinstein Z.B., Manning L. (2020). Sex Mediates Relationships Between Regional Tau Pathology and Cognitive Decline. Ann. Neurol..

[46] Sundermann E.E., Panizzon M., Chen X., Andrews M., Galasko D., Banks S.J. (2020). Sex Differences in Alzheimer’s-Related Tau Biomarkers and the Mediating Effect of Testosterone. Biol. Sex Differ..

[47] Sundermann E.E., Biegon A., Rubin L.H., Lipton R.B., Landau S., Maki P.M. (2017). Alzheimer’s Disease Neuroimaging Initiative Does the Female Advantage in Verbal Memory Contribute to Underestimating Alzheimer’s Disease Pathology in Women versus Men?. J. Alzheimers Dis..

[48] Digma L.A., Madsen J.R., Rissman R.A., Jacobs D.M., Brewer J.B., Banks S.J. (2020). Alzheimer’s Disease Neuroimaging Initiative Women Can Bear a Bigger Burden: Ante- and Post-Mortem Evidence for Reserve in the Face of Tau. Brain Commun..

[49] (2018). Alzheimer’s Association. 2018 Alzheimer’s Disease Facts and Figures. Alzheimer’s Dement..

[50] Gao S., Hendrie H.C., Hall K.S., Hui S. (1998). The Relationships between Age, Sex, and the Incidence of Dementia and Alzheimer Disease: A Meta-Analysis. Arch. Gen. Psychiatry.

[51] Andersen K., Launer L.J., Dewey M.E., Letenneur L., Ott A., Copeland J.R.M., Dartigues J.F., Kragh-Sorensen P., Baldereschi M., Brayne C. (1999). Gender Differences in the Incidence of AD and Vascular Dementia: The EURODEM Studies. Neurology.

[52] Miech R.A., Breitner J.C.S., Zandi P.P., Khachaturian A.S., Anthony J.C., Mayer L. (2002). Incidence of AD May Decline in the Early 90s for Men, Later for Women: The Cache County Study. Neurology.

[53] Lin K.A., Choudhury K.R., Rathakrishnan B.G., Marks D.M., Petrella J.R., Doraiswamy P.M. (2015). Marked Gender Differences in Progression of Mild Cognitive Impairment over 8 Years. Alzheimer’s Dement. Transl. Res. Clin. Interv..

[54] Sundermann E.E., Maki P.M., Rubin L.H., Lipton R.B., Landau S., Biegon A. (2016). Female Advantage in Verbal Memory. Neurology.

[55] Caldwell J.Z.K., Berg J.-L., Cummings J.L., Banks S.J. (2017). Moderating Effects of Sex on the Impact of Diagnosis and Amyloid Positivity on Verbal Memory and Hippocampal Volume. Alzheimer’s Res. Ther..

[56] Barnes L.L., Wilson R.S., Bienias J.L., Schneider J.A., Evans D.A., Bennett D.A. (2005). Sex Differences in the Clinical Manifestations of Alzheimer Disease Pathology. Arch. Gen. Psychiatry.

[57] Sundermann E.E., Bondi M.W., Campbell L.M., Gouaux B., Moore R.C., Soontornniyomkij V., Moore D.J. (2021). Distinguishing Amnestic Mild Cognitive Impairment From HIV-Associated Neurocognitive Disorders. J. Infect. Dis..

[58] McKhann G., Drachman D., Folstein M., Katzman R., Price D., Stadlan E.M. (1984). Clinical Diagnosis of Alzheimer’s Disease: Report of the NINCDS-ADRDA Work Group under the Auspices of Department of Health and Human Services Task Force on Alzheimer’s Disease. Neurology.

[59] Huff F.J., Becker J.T., Belle S.H., Nebes R.D., Holland A.L., Boller F. (1987). Cognitive Deficits and Clinical Diagnosis of Alzheimer’s Disease. Neurology.

[60] Morgello S., Gelman B.B., Kozlowski P.B., Vinters H.V., Masliah E., Cornford M., Cavert W., Marra C., Grant I., Singer E.J. (2001). The National NeuroAIDS Tissue Consortium: A New Paradigm in Brain Banking with an Emphasis on Infectious Disease. Neuropathol. Appl. Neurobiol..

[61] Cysique L.A., Franklin D., Abramson I., Ellis R.J., Letendre S., Collier A., Marra C., Clifford D., Gelman B., McArthur J. (2011). Normative Data and Validation of a Regression Based Summary Score for Assessing Meaningful Neuropsychological Change. J. Clin. Exp. Neuropsychol..

[62] Heaton R.K., Miller S.W., Taylor M.J., Grant I. (2004). Revised Comprehensive Norms for an Expanded Halstead-Reitan Battery: Demographically Adjusted Neuropsychological Norms for African American and Caucasian Adults Scoring Program.

[63] Norman M.A., Moore D.J., Taylor M., Franklin D., Cysique L., Ake C., Lazarretto D., Vaida F., Heaton R.K. (2011). HNRC Group Demographically Corrected Norms for African Americans and Caucasians on the Hopkins Verbal Learning Test-Revised, Brief Visuospatial Memory Test-Revised, Stroop Color and Word Test, and Wisconsin Card Sorting Test 64-Card Version. J. Clin. Exp. Neuropsychol..

[64] Heaton R.K., Grant I., Butters N., White D.A., Kirson D., Atkinson J.H., McCutchan J.A., Taylor M.J., Kelly M.D., Ellis R.J. (1995). The HNRC 500--Neuropsychology of HIV Infection at Different Disease Stages. HIV Neurobehavioral Research Center. J. Int. Neuropsychol. Soc..

[65] Carey C.L., Woods S.P., Gonzalez R., Conover E., Marcotte T.D., Grant I., Heaton R.K. (2004). HNRC Group Predictive Validity of Global Deficit Scores in Detecting Neuropsychological Impairment in HIV Infection. J. Clin. Exp. Neuropsychol..

[66] Blackstone K., Moore D.J., Franklin D.R., Clifford D.B., Collier A.C., Marra C.M., Gelman B.B., McArthur J.C., Morgello S., Simpson D.M. (2012). Defining Neurocognitive Impairment in HIV: Deficit Scores versus Clinical Ratings. Clin. Neuropsychol..

[67] Galasko D., Hansen L.A., Katzman R., Wiederholt W., Masliah E., Terry R., Hill L.R., Lessin P., Thal L.J. (1994). Clinical-Neuropathological Correlations in Alzheimer’s Disease and Related Dementias. Arch. Neurol..

[68] Salmon D., Butters N., Katzman R., Rowe J.W. (1992). Neuropsychological Assessment of Dementia in the Elderly. Principles of Geriatric Neurology.

[69] Duyckaerts C., Delatour B., Potier M.-C. (2009). Classification and Basic Pathology of Alzheimer Disease. Acta Neuropathol..

[70] Soontornniyomkij V., Moore D.J., Gouaux B., Soontornniyomkij B., Tatro E.T., Umlauf A., Masliah E., Levine A.J., Singer E.J., Vinters H.V. (2012). Cerebral β-Amyloid Deposition Predicts HIV-Associated Neurocognitive Disorders in *APOE* Ε4 Carriers. AIDS.

[71] Alafuzoff I., Arzberger T., Al-Sarraj S., Bodi I., Bogdanovic N., Braak H., Bugiani O., Del-Tredici K., Ferrer I., Gelpi E. (2008). Staging of Neurofibrillary Pathology in Alzheimer’s Disease: A Study of the BrainNet Europe Consortium. Brain Pathol..

[72] Adams K.M., Brown G.G., Grant I. (1985). Analysis of Covariance as a Remedy for Demographic Mismatch of Research Subject Groups: Some Sobering Simulations. J. Clin. Exp. Neuropsychol..

[73] Grothe M.J., Barthel H., Sepulcre J., Dyrba M., Sabri O., Teipel S.J. (2017). Alzheimer’s Disease Neuroimaging Initiative In Vivo Staging of Regional Amyloid Deposition. Neurology.

[74] Bowman G.L., Kaye J.A., Moore M., Waichunas D., Carlson N.E., Quinn J.F. (2007). Blood-Brain Barrier Impairment in Alzheimer Disease: Stability and Functional Significance. Neurology.

[75] Nath A. (2002). Human Immunodeficiency Virus (HIV) Proteins in Neuropathogenesis of HIV Dementia. J. Infect. Dis..

[76] Masliah E., Achim C.L., Ge N., DeTeresa R., Terry R.D., Wiley C.A. (1992). Spectrum of Human Immunodeficiency Virus-Associated Neocortical Damage. Ann. Neurol..

[77] Plessis S.D., Vink M., Joska J.A., Koutsilieri E., Stein D.J., Emsley R. (2014). HIV Infection and the Fronto-Striatal System: A Systematic Review and Meta-Analysis of FMRI Studies. AIDS.

[78] Ellis R., Langford D., Masliah E. (2007). HIV and Antiretroviral Therapy in the Brain: Neuronal Injury and Repair. Nat. Rev. Neurosci..

[79] Ernst T., Chang L., Jovicich J., Ames N., Arnold S. (2002). Abnormal Brain Activation on Functional MRI in Cognitively Asymptomatic HIV Patients. Neurology.

[80] Israel S.M., Hassanzadeh-Behbahani S., Turkeltaub P.E., Moore D.J., Ellis R.J., Jiang X. (2019). Different Roles of Frontal versus Striatal Atrophy in HIV-associated Neurocognitive Disorders. Hum. Brain Mapp..

[81] Towgood K.J., Pitkanen M., Kulasegaram R., Fradera A., Kumar A., Soni S., Sibtain N.A., Reed L., Bradbeer C., Barker G.J. (2012). Mapping the Brain in Younger and Older Asymptomatic HIV-1 Men: Frontal Volume Changes in the Absence of Other Cortical or Diffusion Tensor Abnormalities. Cortex.

[82] Sanford R., Fernandez Cruz A.L., Scott S.C., Mayo N.E., Fellows L.K., Ances B.M., Collins D.L. (2017). Regionally Specific Brain Volumetric and Cortical Thickness Changes in HIV-Infected Patients in the HAART Era. J. Acquir. Immune Defic. Syndr..

[83] Guha D., Wagner M.C.E., Ayyavoo V. (2018). Human Immunodeficiency Virus Type 1 (HIV-1)-Mediated Neuroinflammation Dysregulates Neurogranin and Induces Synaptodendritic Injury. J. Neuroinflamm..

[84] Cysique L.A., Moffat K., Moore D.M., Lane T.A., Davies N.W.S., Carr A., Brew B.J., Rae C. (2013). HIV, Vascular and Aging Injuries in the Brain of Clinically Stable HIV-Infected Adults: A (1)H MRS Study. PLoS ONE.

[85] Tan C.-C., Zhang X.-Y., Tan L., Yu J.-T. (2018). Tauopathies: Mechanisms and Therapeutic Strategies. J. Alzheimers Dis..

[86] Bell J.E., Arango J.-C., Anthony I.C. (2006). Neurobiology of Multiple Insults: HIV-1-Associated Brain Disorders in Those Who Use Illicit Drugs. J. Neuroimmune Pharmacol..

[87] Donohue M.C., Sperling R.A., Petersen R., Sun C.-K., Weiner M.W., Aisen P.S. (2017). Alzheimer’s Disease Neuroimaging Initiative Association Between Elevated Brain Amyloid and Subsequent Cognitive Decline Among Cognitively Normal Persons. JAMA.

[88] Steenland K., Zhao L., Goldstein F., Cellar J., Lah J. (2014). Biomarkers for Predicting Cognitive Decline in Those with Normal Cognition. J. Alzheimers Dis..

[89] Tomassen J., den Braber A., van der Landen S.M., Konijnenberg E., Teunissen C.E., Vermunt L., de Geus E.J.C., Boomsma D.I., Scheltens P., Tijms B.M. (2022). Abnormal Cerebrospinal Fluid Levels of Amyloid and Tau Are Associated with Cognitive Decline over Time in Cognitively Normal Older Adults: A Monozygotic Twin Study. Alzheimers Dement.

[90] Salmon D.P. (2011). Neuropsychological Features of Mild Cognitive Impairment and Preclinical Alzheimer’s Disease. Current Topics in Behavioral Neurosciences.

[91] Farrell K., Iida M.A., Cherry J.D., Casella A., Stein T.D., Bieniek K.F., Walker J.M., Richardson T.E., White C.L., Alvarez V.E. (2022). Differential Vulnerability of Hippocampal Subfields in Primary Age-Related Tauopathy and Chronic Traumatic Encephalopathy. J. Neuropathol. Exp. Neurol..

[92] Ossenkoppele R., Schonhaut D.R., Schöll M., Lockhart S.N., Ayakta N., Baker S.L., O’Neil J.P., Janabi M., Lazaris A., Cantwell A. (2016). Tau PET Patterns Mirror Clinical and Neuroanatomical Variability in Alzheimer’s Disease. Brain.

[93] Lowe V.J., Wiste H.J., Senjem M.L., Weigand S.D., Therneau T.M., Boeve B.F., Josephs K.A., Fang P., Pandey M.K., Murray M.E. (2018). Widespread Brain Tau and Its Association with Ageing, Braak Stage and Alzheimer’s Dementia. Brain.

[94] Mattson M.P., Haughey N.J., Nath A. (2005). Cell Death in HIV Dementia. Cell Death Differ..

[95] Hategan A., Bianchet M.A., Steiner J., Karnaukhova E., Masliah E., Fields A., Lee M.-H., Dickens A.M., Haughey N., Dimitriadis E.K. (2017). HIV Tat Protein and Amyloid-β Peptide Form Multifibrillar Structures That Cause Neurotoxicity. Nat. Struct. Mol. Biol..

[96] András I.E., Rha G., Huang W., Eum S., Couraud P.-O., Romero I.A., Hennig B., Toborek M. (2008). Simvastatin Protects against Amyloid β and HIV-1 Tat-Induced Promoter Activities of Inflammatory Genes in Brain Endothelial Cells. Mol. Pharmacol..

[97] Sun B., Fernandes N., Pulliam L. (2019). Profile of Neuronal Exosomes in HIV Cognitive Impairment Exposes Sex Differences. AIDS.

[98] Basso M.R., Bornstein R.A. (2000). Estimated Premorbid Intelligence Mediates Neurobehavioral Change in Individuals Infected with HIV across 12 Months. J. Clin. Exp. Neuropsychol..

[99] Farinpour R., Miller E.N., Satz P., Selnes O.A., Cohen B.A., Becker J.T., Skolasky R.L., Visscher B.R. (2003). Psychosocial Risk Factors of HIV Morbidity and Mortality: Findings from the Multicenter AIDS Cohort Study (MACS). J. Clin. Exp. Neuropsychol..

[100] Tsai A.C., Burns B.F.O. (2015). Syndemics of Psychosocial Problems and HIV Risk: A Systematic Review of Empirical Tests of the Disease Interaction Concept. Soc. Sci. Med..

[101] Singer M. (1994). AIDS and the Health Crisis of the U.S. Urban Poor; the Perspective of Critical Medical Anthropology. Soc. Sci. Med..

[102] Sundermann E.E., Heaton R.K., Pasipanodya E., Moore R.C., Paolillo E.W., Rubin L.H., Ellis R., Moore D.J. (2018). Sex Differences in HIV-Associated Cognitive Impairment. AIDS.

[103] Rubin L.H., Cook J.A., Weber K.M., Cohen M.H., Martin E., Valcour V., Milam J., Anastos K., Young M.A., Alden C. (2015). The Association of Perceived Stress and Verbal Memory Is Greater in HIV-Infected versus HIV-Uninfected Women. J. Neurovirol..

[104] Rubin L.H., Cook J.A., Springer G., Weber K.M., Cohen M.H., Martin E.M., Valcour V.G., Benning L., Alden C., Milam J. (2017). Perceived and Post-Traumatic Stress Are Associated with Decreased Learning, Memory, and Fluency in HIV-Infected Women. AIDS.

[105] Akiyama H., Barger S., Barnum S., Bradt B., Bauer J., Cole G.M., Cooper N.R., Eikelenboom P., Emmerling M., Fiebich B.L. (2000). Inflammation and Alzheimer’s Disease. Neurobiol. Aging.

[106] Borrajo A., Spuch C., Penedo M.A., Olivares J.M., Agís-Balboa R.C. (2021). Important Role of Microglia in HIV-1 Associated Neurocognitive Disorders and the Molecular Pathways Implicated in Its Pathogenesis. Ann. Med..

[107] Stuart K.E., Padgett C. (2020). A Systematic Review of the Association Between Psychological Stress and Dementia Risk in Humans. J. Alzheimers Dis..

