# Supplementary material for: Alzheimer’s Disease Pathology in Middle Aged and Older People with HIV: Comparisons with Non-HIV Controls on a Healthy Aging and Alzheimer’s Disease Trajectory and Relationships with Cognitive Function
Source: Viruses. 2023 Jun 4;15(6):1319. doi: 10.3390/v15061319 (PMC10305373; doi:10.3390/v15061319)
Supplement: Supplementary file 1 [file viruses-15-01319-s001.zip › viruses-2403789-supplementary.pdf]

SUPPLEMENTARY TABLES.

Supplementary Table 1. Comparison of characteristics of PWH sample by sex.

	Women with HIV (N=10)	Men with HIV (N=39)	Test Statistic, p-value
**Demographics**			
Age, *mean (SD)*	56.10 (5.04)	57.69 (5.06)	F(1,47)=0.79, p=.38
Education years, *mean (SD)*	11.60 (2.17)	13.00 (3.05)	F(1,43)=1.83, p=.18
Race/ethnicity			
White, N (%)	2 (20%)	26 (67%)	X^2=7.08, p=.008
Black, N (%)	5 (50%)	10 (26%)	X^2=2.22, p=.14
Hispanic, N (%)	3 (30%)	3 (8%)	X^2=3.69, p=.05
Other, N (%)	0 (0%)	0 (0%)	NA
APOE ε4 carrier, N (%)	2 (20.0%)	9 (24.0%)	X^2=0.05, p=.83
WRAT-3 Reading Subtest, *mean (SD)*	40.2 (9.3)	50.0 (8.6)	F(1,41)=9.50, p=.004
**Antemortem Clinical Comorbidities**			
History of alcohol use disorder, N (%)	6 (60%)	22 (61%)	X^2=0.004, p=.97
History of substance use disorder, N (%)	7 (70%)	23 (66%)	X^2=0.02, p=.88
Major depressive disorder, N (%)	8 (80%)	24 (67%)	X^2=0.20, p=.66
Hypertension, N (%)	3 (38%)	15 (54%)	X^2= 0.32, p=.57
Diabetes, N (%)	0 (0%)	5 (18%)	X^2= 1.43, p=.23
Hyperlipidemia, N (%)	0 (0%)	8 (30%)	X^2=2.37, p=.12
**HIV Disease Characteristics**			
Nadir CD4+ T-cell count (cells/µl), *mean (SD)*	100.29 (65.59)	97.17 (154.65)	F(1,29)=0.00, p=.96
Antemortem CD4+ T-cell count (cells/µl), *mean (SD)*	227.44 (260.02)	192.10 (230.46)	F(1,46)=0.16, p=0.69
Antemortem detectable plasma HIV-1 RNA load (>50 copies/mL), *N (%)*	6 (60.0%)	26 (66.7%)	X^2=0.16, p=.69
Duration of HIV disease, years, *mean (SD)*	12.60 (5.38)	15.32 (6.97)	F(1,46)=1.30, p=.26

Antemortem ART, N (% prescribed)	6 (86%)	28 (85%)	X^2= 0.00 p=.98

Note. PWH = people with HIV. ART = antiretroviral therapy. *APOE* ε4 = apolipoprotein E ε4 allele. WRAT-3 = Wide Range Achievement Test-version 3. Sex differences in sample characteristics were tested using chi-square tests for categorical variables and univariate analyses of variance (ANOVA) for normally-distributed continuous variables.

Supplementary Table 2. Comparison of the Alzheimer's disease pathology positivity rates between women and men with HIV.

	Women with HIV	Men with HIV	Chi-square, p-value, effect size (φ)
**Aβ positivity*, N (%)**			
Frontal neocortex	3 of 10 (30.0%)	17 of 39 (43.6%)	X^2=0.6, p=.43, φ=0.11
Basal temporal neocortex	5 of 8 (62.5%)	7 of 26 (26.9%)	X^2=3.4, p=.07, φ=-0.32
Hippocampus	2 of 7 (28.6%)	4 of 25 (16.0%)	X^2=0.6, p=.45, φ=-0.13
Any brain region	5 of 8 (62.8%)	18 of 31 (58.1%)	X^2=.05, p=.82, φ=-0.04
Widespread Aβ (any brain region)	3 of 8 (37.5%)	9 of 28 (32.1%)	X^2=.08, p=.78, φ=-0.05
**P-Tau positivity*, N (%)**			
Frontal neocortex	8 of 10 (80.0%)	22 of 39 (56.4%)	X^2=1.9, p=.17, φ=-0.20
Basal temporal neocortex	6 of 8 (75.0%)	18 of 26 (69.2%)	X^2=0.1, p=.75, φ=-0.05
Transentorhinal cortex	6 of 7 (85.7%)	16 of 23 (69.6%)	X^2=0.7, p=.40, φ=-0.15
Entorhinal cortex	5 of 7 (71.4%)	9 of 23 (39.1%)	X^2=2.2, p=.13, φ=-0.13
Hippocampus	6 of 7 (85.7%)	16 of 25 (64.0%)	X^2=1.2, p=.27, φ=-0.19
Any brain region[¥]	9 of 9 (100%)	32 of 33 (96.9%)	X^2=0.28, p=.60, φ=-0.08
Widespread p-tau (any brain region)	4 of 7 (57.1%)	11 of 24 (45.8%)	X^2=1.2, p=.27, φ=-0.10

Note. *Pathology positive was defined as pathology grade ≥ 1 with the density of Aβ plaques graded as 0 (absent), 1 (focal), or 2 (widespread), and the density of p-Tau neuropil threads graded as 0 (absent), 1 (barely present at 100x magnification), 2 (easily noted at 100x magnification), or 3 (notable with naked eye inspection). [¥] Comparison is not possible as 100% of cases were p-tau positive in at-least one brain region. Effect size for differences in proportions is measured by the phi coefficient (φ): 0.1 = small, 0.3 = medium, 0.5 = large.

Supplementary Table 3. Comparison of memory performance (demographically-adjusted T-scores) between pathology positive (+) versus pathology negative (-) PWH in sex-stratified samples.

	Learning			Recall		
	Pathology+ Mean (SD)	Pathology- Mean (SD)	ANOVA results	Pathology+ Mean (SD)	Pathology- Mean (SD)	ANOVA results
**Women**						
**Aβ**						
Any brain region	38.6 (8.2)	47.2 (1.5)	F(1,6)=3.0, p=.13, *d*=1.46	40.3 (6.2)	47.7 (3.3)	F(1,6)=3.5, p=.11, *d*=1.49
Widespread Aβ (any brain region)	34.3 (8.1)	46.3 (1.6)	F(1,6)=11.4, **p=.01,** *d*=2.06	36.5 (4.8)	47.0 (2.5)	F(1,6)=3.1, p=.09, *d*=2.74
**P-Tau**						
Any brain region[¥]	NA	NA	NA	NA	NA	NA
Widespread p-tau (any brain region)	37.5 (9.0)	47.2 (1.5)	F(1,5)=3.2, p=.13, *d*=1.50	40.0 (7.1)	47.7 (3.3)	F(1,5)=2.9, p=.15, *d*=1.39
**Men**						
**Aβ**						
Any brain region	39.2 (7.1)	46.4 (11.8)	F(1,27)=4.1, **p=.05,** *d*=0.74	38.2 (9.8)	44.7 (10.7)	F(1,27)=2.9, p=.10, *d*=0.63
Widespread Aβ (any brain region)	38.8 (9.1)	44.5 (10.2)	F(1,24)=1.7, p=.21, *d*=0.59	35.6 (9.9)	43.5 (10.2)	F(1,24)=3.1, p=.09, *d*=0.79
**P-Tau**						
Any brain region[¥]	NA	NA	NA	NA	NA	NA
Widespread p-tau (any brain region)	41.9 (7.5)	44.9 (13.0)	F(1,21)=0.4, p=.52, *d*=0.28	40.7 (10.4)	43.4 (11.8)	F(1,21)=0.3, p=.56, *d*=0.24

Note. Means and standard deviations (SD) are from domain-specific, demographically-adjusted T-scores. Pathology positive was defined as pathology grade ≥ 1 with the density of Aβ plaques graded as 0 (absent), 1 (focal), or 2 (widespread), and the density of p-Tau neuropil threads graded as 0 (absent), 1 (barely present at 100x magnification), 2 (easily noted at 100x magnification), or 3 (notable with naked eye inspection). [¥] Comparison is not possible as 100% of cases were p-tau positive in at-least one brain region.

Supplementary Table 4. Comparison of performance in non-memory cognitive domains (demographically-adjusted domain T-scores) between pathology positive (+) versus pathology negative (-) PWH in sex-stratified samples.

	Executive Function			Speed of Information Processing			Attention/Working Memory			Verbal Fluency			Motor		
	Pathology+ Mean (SD)	Pathology- Mean (SD)	ANOVA results	Pathology+ Mean (SD)	Pathology- Mean (SD)	ANOVA results	Pathology+ Mean (SD)	Pathology- Mean (SD)	ANOVA results	Pathology+ Mean (SD)	Pathology-* Mean (SD)	ANOVA results	Pathology+ Mean (SD)	Pathology- Mean (SD)	ANOVA results
**Women**															
**Aβ**															
Any brain region	47.7 (10.7)	56.8 (14.9)	F(1,6)=1.0, p=.35, *d*=0.70	46.3 (6.3)	42.9 (6.0)	F(1,6)=0.6, p=.48, *d*=0.55	49.5 (3.5)	44.8 (6.3)	F(1,6)=1.9, p=.22, *d*=0.92	51.4 (7.2)	53.0 (10.4)	F(1,6)=0.1, p=.80, *d*=0.18	45.2 (7.7)	50.7 (3.9)	F(1,5)=0.9, p=.40, *d*=0.90
Widespread Aβ (any brain region)	41.3 (5.5)	57.0 (11.5)	F(1,6)=4.6, p=.07, *d*=1.74	43.1 (5.4)	46.2 (6.6)	F(1,6)=0.5, p=.52, *d*=0.51	50.0 (1.0)	46.4 (6.0)	F(1,6)=1.1, p=.35, *d*=0.84	47.7 (7.2)	54.6 (7.7)	F(1,6)=1.6, p=.25, *d*=0.93	47.2 (8.5)	46.5 (7.1)	F(1,6)=0.01, p=.91, *d*=0.09
**P-Tau**															
Any brain region[¥]	NA	NA	NA	NA	NA	NA	NA	NA	NA	NA	NA	NA	NA	NA	NA
Widespread p-tau (any brain region)	48.6 (12.1)	56.8 (14.9)	F(1,5)=0.7, p=.45, *d*=0.60	47.7 (6.3)	42.9 (5.9)	F(1,5)=1.0, p=.36, *d*=0.79	49.6 (4.0)	44.8 (6.3)	F(1,5)=1.5, p=.27, *d*=0.91	53.2 (6.8)	53.0 (10.4)	F(1,5)=0.001, p=.97, *d*=0.02	46.9 (7.8)	50.7 (3.9)	F(1,5)=0.4, p=.56, *d*=0.62
**Men**															
**Aβ**															
Any brain region	55.6 (12.7)	52.6 (12.0)	F(1,27)=0.4, p=.51, *d*=0.24	44.2 (11.4)	50.7 (12.5)	F(1,25)=2.0, p=.17, *d*=0.54	45.6 (10.6)	49.0 (7.2)	F(1,27)=1.0, p=.33, *d*=0.38	46.2 (9.3)	54.5 (13.7)	F(1,27)=3.7, p=.06, *d*=0.71	37.0 (8.8)	40.3 (9.6)	F(1,27)=0.9, p=.36, *d*=0.36
Widespread Aβ (any	57.2 (15.2)	53.1 (12.2)	F(1,24)=0.5, p=.48, *d*=0.3	46.0 (8.7)	49.8 (11.7)	F(1,22)=0.5, p=.48, *d*=0.37	39.8 (7.7)	49.8 (8.5)	F(1,24)=7.4, p=.01, *d*=1.23	43.1 (9.8)	53.0 (12.7)	F(1,24)=3.4, p=.08, *d*=0.87	35.7 (9.0)	39.9 (8.3)	F(1,24)=1.1, p=.32, *d*=0.49

brain region)															
**P-Tau**															
Any brain region[¥]	NA	NA	NA	NA	NA	NA	NA	NA	NA	NA	NA	NA	NA	NA	NA
Widespread p-tau (any brain region)	52.7 (11.1)	54.4 (15.8)	F(1,21)=0.1, p=.77, *d*=0.12	48.9 (9.3)	51.4 (12.7)	F(1,19)=0.3, p=.61, *d*=0.22	48.3 (9.3)	47.4 (10.1)	F(1,21)=0.05, p=.83, *d*=0.09	50.6 (8.3)	51.4 (16.4)	F(1,21)=0.02, p=.88, *d*=0.06	39.7 (9.5)	40.0 (7.1)	F(1,21)=0.01, p=.93, *d*=0.04

Note. Means and standard deviations (SD) are from domain-specific, demographically-adjusted T-scores. Pathology positive was defined as pathology grade ≥ 1 with the density of Aβ plaques graded as 0 (absent), 1 (focal), or 2 (widespread), and the density of p-Tau neuropil threads graded as 0 (absent), 1 (barely present at 100x magnification), 2 (easily noted at 100x magnification), or 3 (notable with naked eye inspection). [¥] Comparison is not possible as 100% of cases were p-tau positive in at-least one brain region.

Supplementary Table 5. Summary of sex-stratified results examining relationships between Aβ and p-Tau positivity status and domain-specific cognitive function.

Cognitive Domain	Aβ pathology (any brain region)<br><br>effect size (statistical significance)	Wide-spread Aβ pathology (any brain region),<br><br>effect size (statistical significance)	p-Tau pathology (any brain region)¥,<br><br>effect size (statistical significance)	Wide-spread p-Tau pathology (any brain region),<br><br>effect size (statistical significance)
**Women with HIV**				
Learning	**L (-)**	**L (+)**	NA	**L (-)**
Recall	**L (-)**	**L (T)**	NA	**L (-)**
Executive Function	L (-)	**L (T)**	NA	M (-)
Speed of Information Processing	M (-)	M (-)	NA	**L (-)**
Attention/Working Memory	**L (-)**	**L (-)**	NA	**L (-)**
Verbal Fluency	S (-)	**L (-)**	NA	S (-)
Motor	**L (-)**	S (-)	NA	M (-)
**Men with HIV**				
Learning	**L (+)**	M (-)	NA	S (-)
Recall	M (T)	**L (T)**	NA	S (-)
Executive Function	S (-)	S (-)	NA	S (-)
Speed of Information Processing	M (-)	M (-)	NA	S (-)
Attention/Working Memory	M (-)	**L (+)**	NA	S (-)
Verbal Fluency	**L (T)**	**L (T)**	NA	S (-)
Motor	S (-)	M (-)	NA	S (-)

Note. Cells represent Cohen's *d* effect size for relationships between pathology positivity status (yes/no) and domain-specific cognitive function: L= large effect size, M = moderate effect size, S = small effect size. Large (strong) relationships are bolded. Effect sizes are reported and guided the interpretation of results in the sex-stratified analyses due to the small sample size of women with HIV and the sample size disparity between men and women with HIV. (+) indicates a significant association at $p<.05$, (T) indicates a relationship that is a statistical trend, $p\leq.10$ and (-) indicates a relationship that is not significant or a trend. ¥ Comparison is not possible as 100% of cases were p-tau positive in at-least one brain region.
